# Sex-Specific Changes in Physical Performance Following Military Training: A Systematic Review

**DOI:** 10.1007/s40279-018-0983-4

**Published:** 2018-09-19

**Authors:** Jo Varley-Campbell, Chris Cooper, Daryl Wilkerson, Sophie Wardle, Julie Greeves, Theo Lorenc

**Affiliations:** 1 Department of Clinical, Educational and Health Psychology, University College London, London, WC1E 7HB UK; 2 Sport and Health Science, University of Exeter, Devon, EX1 2LU UK; 3 Army Personnel Research Capability, Army Headquarters, Andover, Hampshire SP11 8HT UK; 4 London, UK

## Abstract

**Introduction:**

Men and women joining the military undergo the same training, often in mixed-sex platoons. Given the inherent physiological and physical performance differences between men and women, it is reasonable to question whether sex differences exist in the adaptation to military training and, therefore, whether sex-specific training should be employed to optimise training adaptations.

**Objective:**

To systematically review the literature evaluating changes in the physical performance of men and women following military training.

**Methods:**

Six database sources were searched in addition to extensive secondary searching. Primary prospective intervention studies (all designs) evaluating physical training interventions in military populations, reporting pre- to post-training changes in physical fitness outcomes for both women and men, were included.

**Results:**

We screened 3966 unique records. Twenty-nine studies (*n* = 37 study reports) were included, most of which were conducted in the USA and evaluated initial training for military recruits. Positive changes were more consistently observed in aerobic fitness and muscle strength (whole body and upper body) outcomes than lower body strength, muscle power or muscle endurance outcomes, following physical training. Relative pre- to post-training changes for all outcome measures tended to be greater in women than men although few statistically significant sex by outcome/time interactions were observed.

**Conclusion:**

Improvements in some, but not all, performance components were observed following a period of military training. Largely, these improvements were not significantly different between sexes. Further prospective research is needed to evaluate sex-specific differences in the response to physical training in controlled conditions to improve military physical training outcomes for both sexes.

**Electronic supplementary material:**

The online version of this article (10.1007/s40279-018-0983-4) contains supplementary material, which is available to authorized users.

## Key Points

**Table:** 

Some aspects of physical performance are improved following military training in both military men and women.
Typically, there were no sex differences in the physical performance adaptation to military training.
It seems sex-specific military training may not be necessary to achieve some improvements in physical performance.

## Introduction

Preparation of personnel for military roles begins with an initial phase of basic military training (BMT), typically ranging from 6 to 14 weeks (depending on arm of service/nation), followed by a period of specialist ‘trade’ training. The purpose of BMT is to transform a civilian into a trained soldier, with a focus on field craft, map reading, weapon handling and formal physical training. Women typically train alongside men during BMT, with the exception of standard entrants in the British Army, who, since 2006, have completed identical training courses in single-sex platoons.

Despite men and women undergoing the same BMT, little is currently known about whether men and women adapt in a similar manner to physical training. Given the sex differences in physiology and physical performance [1], and in the physical demands of BMT [2, 3], we may reasonably expect men and women to adapt differently to physical training. Sex differences in the adaptation to military training would highlight a potential need to train men and women differently to optimise training outcomes. Moreover, sex-specific training would have implications for typical delivery of BMT, and, combined with the typically lower performance levels of women, the recent introduction of women into physically arduous Ground Close Combat (GCC) roles across a number of nations including the UK, USA and Australia.

We conducted a systematic review with the primary aim of understanding sex differences in physical performance changes following military training. A secondary aim of the review was to understand the components of fitness developed to the greatest degree during military training, evaluating any sex differences in improvements of these fitness components. Given that the effectiveness of a GCC soldier is underpinned by physical employment standards spanning the range of fitness components, understanding the components of fitness that require greatest focus/represent the greatest sex difference in performance will enable development of training strategies to appropriately prepare women for the demands of GCC employment.

## Methods

This systematic review was undertaken following guidance published by the National Health Service (NHS) Centre for Reviews and Dissemination [4]. This systematic review is reported in accordance with PRISMA reporting guidelines. The protocol for this review is registered with PROSPERO: CRD42016032870.

### Study Identification

The following bibliographic databases were systematically searched in December 2015: MEDLINE and MEDLINE in Process via Ovid; Embase via Ovid; CINAHL via EBSCO; HMIC via Ovid; SPORTDiscus via EBSCO; and Web of Science via Thomson Reuters (including conference proceedings). The search strategy took the following form: (terms for tri-service populations) and (terms for training or physical training) and (terms for men and women). The searches were not limited by language and they were run from database inception, in each case.

The following supplementary search methods were undertaken: web searching [the meta-search engine Dogpile was used and specific websites were hand-searched (e.g. Defence Technical Information Centre)], a search of PubMed [5] restricted to e-publications, and grey literature searching [via Open Grey and integrating grey literature provided by the Defence Science and Technology Laboratory (DSTL)] [6].

All studies included at full-text were forwards citation chased (using Scopus via Elsevier) and backwards citation chased for 1 generation (manually). Where possible, and for studies published after 1999, study authors were contacted to identify any in-process or unpublished studies. Finally, lateral searching on first and last authors was also undertaken (using Scopus via Elsevier).

The approach to study identification from this systematic review is transparently reported in the Electronic Supplementary Material Appendix S1. Study identification was undertaken by CC, a qualified information specialist. All studies identified were loaded into Endnote 7.3 (Thomson Reuters) and de-duplicated. Data were retained in Research Information Systems (RIS) format for each database created.

### Selection of Studies

An initial sample of 10% of abstracts (*n* = 194) were screened independently by three reviewers to pilot the inclusion criteria and ensure consistency prior to undertaking title and abstract screening. Inter-rater agreement was 96.4% and discrepancies were resolved by discussion.

The remaining studies (*n* = 1755) were single-screened. All studies were screened hierarchically based on the exclusion criteria presented in Table 1. Studies were required to report pre/post results following a military training programme in the same military population, and to be prospective in design. Where the title or abstract met the criteria (or if this was unclear), the full text was retrieved and screened. Full-text screening was undertaken by two reviewers. Each full text was second-screened by a third reviewer. Inter-rater agreement was 100%.

**Table 1:** Exclusion criteria

Exclusion code (EX)	Notes
EX1: not primary prospective intervention study in humans	Include any intervention study (randomised trial, non-randomised trial, one-group uncontrolled study) that reports data from both before and after the intervention. Exclude purely observational or retrospective studies (but include where prospectiveness is unclear, if pre–post data are reported.) Exclude reviews and other secondary research (but retain systematic reviews for subsequent reference checking). Exclude animal studies
EX2: not military population	Include any military population
EX3: not aged 17–60 years	Include studies where the sample is entirely aged between 17 and 60 years; or where the mean age of the sample lies between 17 and 60 years; or where separate data on this age group are reported
EX4: not relevant outcome	Include the following outcomes: muscle strength; muscle endurance; muscle power; aerobic capacity; anaerobic capacity; detraining response; injury (e.g. overuse injury, stress fracture, musculoskeletal injury); energy deficit
EX5: not physical training programme	Include any form of physical training or conditioning intervention. Include multi-component interventions with an exercise or physical training component
EX6: systematic reviews	Relevant systematic reviews were kept separate for screening of their included studies
EX7: no data for both men and women, or different interventions for men and women	Exclude studies not reporting pre- and post-data for both men and women within the sample. Exclude studies not using the same outcome measure for men and women. Exclude studies where men and women received clearly different interventions
EX8: not the same measure and sample at pre and post	Exclude studies using different outcome measures at pre and post time points. Exclude studies using different samples (i.e. different individuals, not counting attrition) at pre- and post-time points

Systematic reviews did not satisfy the inclusion criteria for this review. However, any systematic reviews that were of topic relevance were retained and their included studies screened for inclusion in this systematic review.

### Quality Appraisal and Data Extraction

All studies included at full-text were quality-assessed using a modified form of the Effective Public Health Practice Project (EPHPP) tool for quantitative outcome studies [7].

Study data were extracted by one reviewer and checked by a second reviewer, using a standardised form that included information on selection bias, study design, confounders, blinding, data collection methods and withdrawals and dropouts. These sub-domains were considered along with intervention integrity and analysis methods to give an overall rating for the study quality. Studies could be rated as providing either weak, moderate or strong quality evidence.

### Statistical Analysis and Data Synthesis

The studies were synthesised descriptively. Outcome measures were categorised initially into two overarching categories (‘aerobic fitness’ and ‘strength and muscular endurance’) and then into narrower categories within these two overarching categories (e.g. maximal oxygen uptake ($\dot{V}{\text{O}}_{2\text{max} }$), run time, whole body strength/power, muscle endurance, push-ups, sit-ups, upper/lower body strength, grip strength). Due to the limited validity of the studies, and, in particular, the few controlled studies, a full meta-analysis could not be undertaken. Where data allowed and outcomes were similar, a graphical format was used to summarise the change between pre- and post-training and, if reported, any statistical significance of this change (as reported in the included studies by their authors). Where this approach was not possible, data were presented in tabular form. The tables report the pre- and post-training results along with calculated relative percentage change and any significant changes (as reported in the included studies by their authors). Relative percentage change was calculated as ((post score − pre score)/(pre score)). Standardised gain scores were not calculated as these may have been unreliable for within-subject designs where individual participant data were unavailable. Moreover, reporting absolute values and percentage changes allows for a more intuitive interpretation of the magnitude of the observed changes. To provide a summary of the observed changes, the median of the pre–post changes observed in each study was taken, un-weighted by sample size or standard deviation. This method provides an indication of the approximate magnitude of the observed changes, but should not be regarded as a pooled effect size, and in some cases it subsumes heterogeneous outcome measures.

## Results

### Results of Searches

**Fig. 1:**
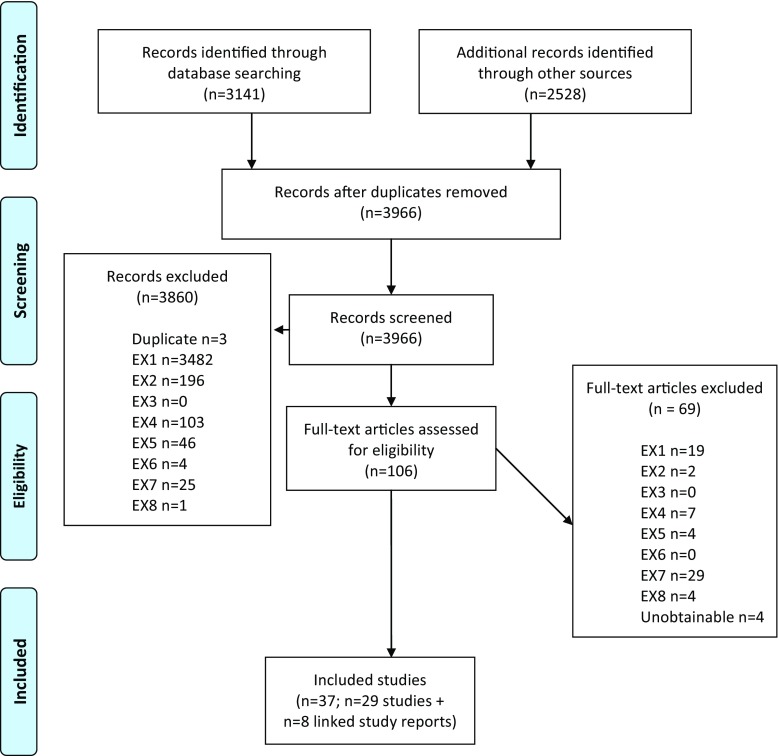
PRISMA study selection flow chart. *EX1* not primary prospective intervention in humans, *EX2* not military population, *EX3* not aged 17–60 years, *EX4* not relevant outcomes, *EX5* not physical training programme, *EX6* systematic review, *EX7* no data for both men and women individually, *EX8* not the same measure and sample for pre/post

A total of 3966 citations were identified by our searches. 106 studies (2.7% of the total studies identified) were taken forward to full-text screening and 29 studies have been included in this systematic review with an additional eight linked study reports (in total 37 included citations or 0.9% of the original citations identified from the search). Three systematic reviews (Knapik et al. [8], Wentz et al. [9] and Jones et al. [10]) and one meta-analysis (Courtright et al. [11]) were identified^1^ and their included studies screened for inclusion in this review. The PRISMA diagram is shown in Fig. 1.

### Study Characteristics

Of the 29 included studies [2, 3, 12–38], 24 utilised a one-group pre–post design [2, 3, 12–16, 18–21, 23–25, 27–35, 37], three studies reported a two-group pre–post design [22, 26, 36], one was a randomised controlled trial [17] and one was a non-randomised controlled trial [38]. Six of the studies were conducted in the UK [2, 3, 19, 25, 28, 35], 14 in the USA [12–14, 21–24, 27, 29–33], three in Israel [16, 37, 38], two in Australia [15, 26], two in Canada [18, 20], one in South Africa [36] and one in Germany [34]. The training intervention was typically (*n* = 24 studies) a basic combat/recruit training programme of the country of study. Three studies [21, 22, 36] reported a comparison between standard basic training and an altered version of basic training. One study [17] reported a comparison between an exercise programme and a combined exercise and diet programme. Finally, one study [24] reported changes following a circuit-based weight-training programme. Study durations ranged between 6 and 14 weeks, except for Harwood et al. (40 weeks [19]) and Daniels et al. (23 months [14]). Outcomes reported included measures of aerobic and anaerobic fitness tests, muscle strength (whole, upper and lower body), muscle endurance, whole-body power, grip strength and flexibility. Study characteristics are reported in Table 2.

**Table 2:** Study characteristics

Study	Country/service	Total *N*^a^	Sex	*N* by sex^a^	Study duration	Retention	Outcomes	Intervention
Knapik et al. [22]	US Army recruits	2580	Female int	515	9 weeks	NR	2-mile run time, push ups, sit ups, injury rate	Intervention: Physical Readiness Program incorporated into BCT. Including calisthenics, dumbbell drills, movement drills, interval training, long-distance running and flexibility training. The programme was followed for the initial 7 (of 9) weeks of BCT, after which the int group switched to the same programme as the comp group. Comparison: ‘traditional’ BCT physical training including warm-up, stretching, calisthenics, variations on push up and sit up exercises and running in formation in ability groups
			Female comp	651				
			Male int	769				
			Male comp	645				
Teves et al. [32]	US Army recruits	1984	Female	1004	8 weeks	49%	$\dot{V}{\text{O}}_{2\text{max} }$, hand grip strength, upright pull, incremental dynamic lift	8 weeks of BT for phase 1 to phase 2
			Male	980		47%		
Knapik et al. [23]	US Army recruits	1444	Female	496	7 weeks	70%	Upper torso strength, leg extensor strength, trunk extensor strength	Army basic initial entry training; including 39 h of physical activity of calisthenics, strength exercises, running and marching. Calisthenics and strength exercises performed ~ 1 h/day 5–6 days/week
			Male	948		77%		
Knapik et al. [21]	US Army recruits	1138	Female	482	7 weeks	NR	2-mile run time, push ups, sit ups, injury incidence	Intervention: BCT of ~ 1 h physical training each morning, including conditioning drills, movement drills, stretching drills, speed running, ability group running, and shuttle running. Comparison: N/A. Two comparisons are reported but neither provides usable effectiveness data
			Male	656				
Bell et al. [12]	US Army recruits	861	Female	352	8 weeks	NR	Run time, push ups; sit ups, injury incidence	BCT; no further information
			Male	509				
Hart et al. [18]	Canadian recruits	587	Female	278	9 weeks	NR	Incremental lift machine, max static exertion	BCT; no further information
			Male	309				
Yanovich et al. [38]	Israel Defence Force recruits	420	Female combat	221	4 and 16 months	24%	$\dot{V}{\text{O}}_{2\text{max} }$	Army BT for 4 months (and subsequent military service for 1 year). Undertaken in desert-climate conditions and in a gender-integrated battalion
			Female non-combat	121				
			Male combat	78		27%		
Wood and Kruger [36]	South African health and medical service recruits	373	Female int	85	12 weeks	98% (99% int, 96% comp)	Run time, push ups; sit ups, walk time, shuttle runs	Intervention: BMT programme. 48 × 40-min activity sessions over 12 weeks. Total components 322 min warm-up, 950 min jogging, 213 min interval training; 28 × body-weight upper-body endurance exercises, 64 × with 20 kg poles; 28 × body-weight abdominal endurance exercises, 64 × with 20 kg poles. Comparison: BMT as intervention but more time for warm-up and less endurance exercises, and the poles (weights) were not used: 630 min warm-up, 510 min jogging, 200 min interval training; 51 × body-weight upper-body endurance exercises; 56 × body-weight abdominal endurance exercises
			Female comp	115				
			Male int	100				
			Male comp	73				
Sharp et al. [30]	US Army recruits	350	Female	168	8 weeks	60%	$\dot{V}{\text{O}}_{2\text{max} }$, upper torso strength, lower body strength, upright pull, dynamic lifting, vertical jump power, peak power, hamstring flexibility	BCT; no further information
			Male	182		54%		
Vogel et al. [33]	US Army recruits	345	Female	159	6 weeks	NR	$\dot{V}{\text{O}}_{2\text{max} }$, muscle strength	US Army BT
			Male	186				
Evans et al. [16]	Israeli Defence Force	257	Female	199	4 months	77%	$\dot{V}{\text{O}}_{2\text{max} }$	Gender-integrated basic recruit training. Training programme included marching under load, running and jumping, battle drills
			Male	58		71%		
Jetté et al. [20]	Canadian force recruits	211	Female	96	9 weeks^b^	71%	Incremental lift machine, static pull, shoulder arm push, grip strength, bicep curl	BMT included 63 × 40-min periods: 30% walking/jogging/marching and the rest consisting of roughly equal proportions of: physical training exercises, circuit training, sports, swimming and performance testing
			Male	115		66%		
Patton et al. [27]	US Army recruits	200	Female	100	7 weeks	57%	$\dot{V}{\text{O}}_{2\text{max} }$, run time	Army BT; 39 h of physical training over 7 weeks, including: daily runs, marching to field exercises; calisthenics, log exercises, rifle drills
			Male	100		87%		
Sharp et al. [29]	US Army recruits	200	Female 1	100	8 weeks	43%	$\dot{V}{\text{O}}_{2\text{max} }$	BT, no further information
			Female 2					
			Male 1	100				
			Male 2					
Yanovich et al. [37]	Israeli Defence Force recruits	176	Female	129	4 months	84%	$\dot{V}{\text{O}}_{2\text{max} }$, run time, push ups, sit ups, ground reaction force, peak power	BCT over 4 months, including average of 4 h running, 3 h marching, 10 h combat training and 5 h continuous standing per week
			Male	47		60%		
Drain et al. [15]	Australian Army recruits	174	Female	20	7–8 weeks	NR	Max box lift	40 × physical training, each 45–60 min. Including: circuit training (7 sessions), running (6), swimming (3), load carriage (7), obstacle course (4), fitness testing (3), and familiarisation or skill-based sessions (10)
			Male	154				
Sonna et al. [31]	US Army recruits	147	Female	85	8 weeks	72%	$\dot{V}{\text{O}}_{2\text{max} }$	BT: 1–1.5 h, 4–6 days/week. Alternated between aerobic and muscle strength training (typically each 2 sessions/week). Aerobic training: 0.5–3 mile runs, timed according to ability, and sprinting. Strength training: push-ups, sit-ups. In addition, participants took part in road marches, obstacle courses, rappelling, and other physical training activities
			Male	62		90%		
von Restorff [34]	German medical service recruits and temp volunteers	110	Female	62	3 months	89%	Right and left hand grip strength, lift from squat and from standing, press from shoulder level, carrying simulated patient of 60, 70, 80 and 90 kg	BMT, details NR
			Male	48		75%		
Harwood et al. [19]	British Army officer cadets in the Royal Military Academy	106	Female	38	40 weeks	NR	Run time, sit ups, static lift, dynamic lift, back extension, pull ups, progressive run	93 × physical training sessions of 45-min. Term 1: basic fitness and battle training; term 2: endurance and battle training; term 3, preparation for competitions and military exercises. The PT sessions included conditioning (8), endurance training (mainly marching; 40), battle training (mainly assault course; 23), basic training (mainly gym skills; 13), and swimming (8)
			Male	68				
Rayson et al. [28]	British Army recruits	72	Female	28	9 weeks	64%	Run time	CMS(R); no further information
			Male	44				
Patterson et al. [26]	Australian defence force	63	Female	28	12 weeks	43%	$\dot{V}{\text{O}}_{2\text{max} }$, push ups, pull ups, 30 s work, peak power, static lift, right and left hand force, bench press, leg press, run dodge and jump course time	3 × 1 h sessions/week. Intervention group participants were split: those with low muscular strength received an intervention focusing on muscular strength, those with low aerobic fitness one focusing on increasing aerobic capacity. Both consisted of weight training, circuit training, running, pack marches, and box and skip sessions in varying proportions
			Male	35		50%		
Richmond et al. [3]	British Army recruits	60	Female	30	14 weeks	53%	Run time, days lost to injury	CMS(R), no further information
			Male	30		57%		
Daniels et al. [13]	US Army cadets at military academy	60	Female	30	6 weeks	90%	$\dot{V}{\text{O}}_{2\text{max} }$, run time	Initial physical and military training programme prior to start of academic year. Physical training included 30-min run 5–6 ×/week in ability groups; unclear what other physical training was undertaken
			Male	30		97%		
Blacker et al. [2]	British Army recruits	54	Female platoon	19	12 weeks	57%	Run time	CMS(R); no further information
			Male platoon	17		77%		
			Mixed platoon	18 (9 females, 9 males)		NR		
Williams et al. [35]	British Army recruits	52	Female	9	10 weeks	60%	$\dot{V}{\text{O}}_{2\text{max} }$, multi-stage shuttle run, 15 m box lift, repetitive lift and carry, loaded (15 kg) march, isometric 38 cm upright pull, incremental dynamic lift to 145 m	10 weeks of BT with modified physical training (PT) consisting of strength training (28 sessions), endurance training (15) agility (8), material handling (6), sports (6), circuit training (4) and swimming (4)
			Male	43				
Marcinik and Hodgdon [24]	US Navy	50	Female	15	10 weeks	60%	Shoulder press, bench press, arm curl, lat pull down, one and two arm lift, leg press, knee extension, muscular endurance leg and bench press, max work capacity, sit and reach	Circuit training program performed on a multi-station gym. 3 sessions/week. Working at 40% of 1RM, 5 s work/15 s move to next station. 3 circuits were completed (11 stations). 1RM was re-evaluated after 5 weeks training. Exercises: bench press, shoulder press, hip flexor, pull-up (or leg lift for women), arm-curl, lat pull-down, leg press, knee extension, arm dip, sit up, handgrip
			Male	35		83%		
Mason et al. [25]	British Army recruits	42	Female	20	10 weeks	NR	Run time, ab curl, injuries reported, upright pull, heaves, lift mean power/max power/total work/max force, MSFT	CMS(R). Training included running, marching, strength training and sports. Mean daily distance covered 11.2 km
			Male	22				
Gambera et al. [17]	US Air Force active-duty personnel	32	Female ex	5	90 days	100%	$\dot{V}{\text{O}}_{2\text{max} }$	Intervention: Mandatory exercise program three times a week. Exercise to incorporate large muscle groups at an intensity of 60–80% of max HR for 40 min. Activities included walking, jogging, cycling, and step-aerobic programs. Comparison: Exercise as above, plus weekly individualised dietary counselling from dietician (Note that all groups received same training intervention; comparison not relevant for this review)
			Male ex	12		100%		
			Female ex + diet	7		100%		
			Male ex + diet	8		100%		
Daniels et al. [14]	US Army cadets at military academy	18	Female	7	23 months	NR	$\dot{V}{\text{O}}_{2\text{max} }$, lost time from injury, upright pull strength, upper torso strength, trunk extensor strength, leg extensor strength	2 years of training. Physical training: calisthenics, grass drills and 30-min run in ability groups 5–6 ×/week. Military field training: combat training and survival, physical training. Physical education classes (boxing and wrestling for Males, self-defence for Women) and a sport club
			Male	11				

*ab* abdominal, *BT* basic training, *BCT* basic combat training, *BMT* basic military training, *comp* comparison, *CMS(R)* Common Military Syllabus for Recruits, *ex* exercise, *int* intervention, *h* hour, *HR* heart rate, *la*t lateral, *max* maximum, *MSFT* multi stage fitness test, *N/A* not applicable, *NR* not reported, *PT* personal trainer, *RM* repetition maximum, *temp* temporary, $\dot{V}{\text{O}}_{2\text{max} }$ maximum oxygen uptake

^a^Numbers recruited

^b^Two of six platoons were tested in week 7

Combined, a total of 12,166 participants (5683 women and 6483 men) were recruited to take part in these studies. The largest study recruited 2580 participants [22] and the smallest 18 participants [14]. The mean age of the participants was between 18.6 and 23.4 years, except for Marcinik and Hodgdon [24], Mason et al. [25] and Gambera et al. [17], where the mean age ranged from 27.7 to 33.8 years. Body mass index (BMI), where reported (*n* = 8) [16, 17, 21, 22, 31, 34, 36, 37], ranged from 22.4 to 25.1 kg/m^2^ in women and between 21.1 and 27.1 kg/m^2^ in men. Percentage body fat, where reported (*n* = 14) [2, 3, 12, 13, 16, 23, 24, 27, 28, 31, 32, 34, 35, 37], ranged from 20.0 to 30.8% in women and from 9.5 and 21.1% in men. The sample populations were classified within normal BMI and percentage body fat guidelines for active individuals and therefore indicative of healthy individuals. Baseline characteristics of the participants are reported in Table 3.

**Table 3:** Baseline characteristics (mean ± SD)

Study	Sex	*N* baseline	Age (years)	Height (m)	Weight (kg)	BMI (kg/m^2^)	% body fat
Knapik et al. [22]	Female int	507	20.9 ± 3.7	1.64 ± 0.06	62.0 ± 9.7	23.0 ± 3.1	NR
	Female comp	637	20.7 ± 3.4	1.64 ± 0.06	61.2 ± 9.1	22.9 ± 2.9	NR
	Male int	759	20.9 ± 3.4	1.77 ± 0.07	75.6 ± 13.3	24.3 ± 3.8	NR
	Male comp	630	20.7 ± 3.3	1.76 ± 0.07	74.4 ± 12.6	24.0 ± 3.7	NR
Teves et al. [32]	Female	487	20.1 ± 3.2	1.63 ± 0.06	58.1 ± 6.8	NR	24.7 ± 3.8^a^
	Male	465	19.2 ± 2.2	1.75 ± 0.07	72.4 ± 10.3	NR	16.0 ± 5.0^a^
Knapik et al. [23]	Female	393	20.7 ± 3.2	1.62 ± 0.07	59.1 ± 7.1	NR	28.0 ± 4.7^a^
	Male	769	19.8 ± 2.7	1.74 ± 0.07	70.9 ± 10.6	NR	16.3 ± 5.1^a^
Knapik et al. [21]	Female	482	21.4 ± 4.0	1.63 ± 0.06	62.4 ± 9.7	23.3 ± 3.0	NR
	Male	656	21.9 ± 4.1	1.77 ± 0.07	78.4 ± 13.5	25.1 ± 3.8	NR
Bell et al. [12]	Female	352	20.0 ± NR	1.62 ± 0.06	57.8 ± 6.3	NR	26.6 ± 4.0^b^
	Male	509		1.75 ± 0.07	76.3 ± 12.3	NR	16.4 ± 5.6^b^
Hart et al. [18]	Female	278	Range 17–25	NR	NR	NR	NR
	Male	309		NR	NR	NR	NR
Yanovich et al. [38]	Female combat	221	19.0 ± 0.9	NR	60.6 ± 10.1	NR	NR
	Female non-combat	121	18.6 ± 0.4	NR	57.6 ± 9.5	NR	NR
	Male combat	78	19.2 ± 1.1	NR	69.8 ± 13.1	NR	NR
Wood and Kruger [36]	Female int	85	20.0 ± 3.2	1.59 ± 0.06	60.2 ± 9.0	22.4 ± 2.5	NR
	Female comp	115	19.9 ± 3.1	1.60 ± 0.05	59.1 ± 8.7	22.8 ± 2.8	NR
	Male int	100	20.2 ± 3.3	1.72 ± 0.06	61.8 ± 6.9	21.4 ± 2.2	NR
	Male comp	73	20.5 ± 3.4	1.71 ± 0.06	62.3 ± 6.7	21.1 ± 2.4	NR
Sharp et al. [30]	Female	168	21.4 ± 3.4	1.63 ± 0.06	62.6 ± 9.8	NR	NR
	Male	182	21.8 ± 3.4	1.77 ± 0.07	78.9 ± 12.8	NR	NR
Vogel et al. [33]	Female	159	19.6 ± 2.3	NR	NR	NR	NR
	Male	186	21.1 ± 2.3	NR	NR	NR	NR
Evans et al. [16]	Female	199	19.0 ± 0.9	1.62 ± 0.06	60.8 ± 10.3	23.2 ± 3.4	30.8 ± 4.8^a^
	Male	58	19.2 ± 1.1	1.75 ± 0.07	68.9 ± 13.1	22.4 ± 3.5	17.4 ± 5.0^a^
Jetté et al. [20]^c^	Female	96	19.7 ± 2.0	1.63 ± 0.06	56.6 ± 7.1	NR	53.8 ± 14.8^d^
	Male	115	20.1 ± 2.6	1.75 ± 0.07	68.3 ± 9.8	NR	36.7 ± 14.9^d^
Patton et al. [27]	Female	100	19.7 ± 1.9	1.60 ± 0.06	56.9 ± 6.1	NR	28.2 ± 4.6^a^
	Male	100	19.6 ± 2.0	1.73 ± 0.07	69.6 ± 10.6	NR	16.3 ± 5.0^a^
Sharp et al. [29]	Female 1	20	19.6 ± 1.8	NR	56.7 ± 7.1	NR	NR
	Female 2	24	19.1 ± 1.3	NR	57.3 ± 6.1	NR	NR
	Male 1	22	19.0 ± 1.5	NR	73.4 ± 11.4	NR	NR
	Male 2	20	19.1 ± 2.0	NR	68.2 ± 10.2	NR	NR
Yanovich et al. [37]	Female	108	19.0 ± 1.0	1.62 ± 0.06	60.5 ± 10.0	23.0 ± 3.4	28.6 ± 4.2^a^
	Male	28		1.74 ± 0.07	69.4 ± 12.6	23.7 ± 4.1	17.4 ± 4.9^a^
Drain et al. [15]	Female	20	23.1 ± 4.6	1.66 ± 0.05	64.0 ± 7.4	NR	NR
	Male	154	21.4 ± 4.2	1.79 ± 0.06	77.9 ± 12.1	NR	NR
Sonna et al. [31]	Female	85	21.7 ± 3.6	NR	NR	23.1 ± 3.1	27.9 ± 6.1^e^
	Male	62		NR	NR	24.8 ± 3.0	16.4 ± 5.7^e^
von Restorff [34]	Female	62	20.2 ± 2.4	1.68 ± 0.07	65.3 ± 8.8	23.0 ± 2.8	27.7 ± 4.0^a^
	Male	48	20.5 ± 1.8	1.80 ± 0.08	79.7 ± 13.3	24.5 ± 2.1	17.9 ± 4.4^a^
Harwood et al. [19]	Female	38	23.4 ± 1.7	1.67 ± 0.05	65.5 ± 5.3	NR	NR
	Male	68	22.8 ± 1.4	1.80 ± 0.07	77.9 ± 8.7	NR	NR
Rayson et al. [28]	Female	28	19.5 ± 3.2	1.66 ± 0.05	61.8 ± 7.1	NR	23.0 ± 4.0^f^
	Male	44	20.5 ± 3.5	1.75 ± 0.08	67.7 ± 8.6	NR	10.0 ± 4.0^f^
Patterson et al. [26]	Female	28	NR	NR	NR	NR	78.0 ± 17.8^d^
	Male	35	NR	NR	NR	NR	63.7 ± 26.0^d^
Richmond et al. [3]	Female	30	18.6 ± 1.9	1.63 ± 0.06	57.2 ± 6.5	NR	20.0 ± 3.6^f^
	Male	30	18.9 ± 1.6	1.80 ± 0.07	73.8 ± 12.9	NR	9.5 ± 4.0^f^
Daniels et al. [13]	Female	30	Range 17–21	1.64 ± 0.06	57.7 ± 6.0	NR	23.8 ± 4.0^a^
	Male	30		1.77 ± 0.05	70.6 ± 7.6	NR	13.1 ± 3.2^a^
Blacker et al. [2]	Female platoon	19	20.1 ± 3.4	1.71 ± 0.08	65.3 ± 8.5	NR	15.0 ± 8.0^f^
	Male platoon	17					
	Mixed platoon	18 (9.9)					
Williams et al. [35]	Female	9	19.1 ± 2.2	1.64 ± 0.07	62.0 ± 7.2	NR	24.9 ± 3.2^f^
	Male	43	19.2 ± 2.6	1.76 ± 0.07	73.0 ± 10.6	NR	11.3 ± 2.8^f^
Marcinik and Hodgdon [24]	Female	9	27.7 ± 4.2	1.66 ± 0.05	65.0 ± 9.6	NR	23.5 ± 5.7^b^
	Male	29	33.8 ± 5.5	1.78 ± 0.07	83.1 ± 14.8	NR	21.1 ± 6.3^b^
Mason et al. [25]	Female	20	NR	NR	NR	NR	NR
	Male	22	NR	NR	NR	NR	NR
Gambera et al. [17]	Female ex	5	32.2 ± 7.4	NR	71.6 ± 3.3	25.1 ± 1.0	NR
	Male ex	12	32.8 ± 6.2	NR	77.1 ± 10.7	25.2 ± 2.8	NR
	Female ex + diet	7	32.7 ± 8.3	NR	66.1 ± 6.2	24.0 ± 3.1	NR
	Male ex + diet	8	33.8 ± 7.1	NR	86.9 ± 10.0	27.1 ± 1.6	NR
Daniels et al. [14]	Female	7	NR	NR	NR	NR	NR
	Male	11	NR	NR	NR	NR	NR

*comp* comparison, *DEXA* dual-energy X-ray absorptiometry, *Int* intervention, *NR* not reported

^a^Average of four-site skin fold

^b^Circumference measurements

^c^Two of six platoons were tested in week 7

^d^Sum for four skin folds (mm)

^e^DEXA

^f^Bioelectrical impedance

Most studies measured improvements only up to the end of the training programme, which was between 1.5 and 4 months for all studies [2, 3, 12, 13, 15–18, 20–38], with the exception of two which had longer durations of 40 weeks [19] and 100 weeks [14]. To maximise comparability, measurements collected at the end of the training programme have been used for the analyses below. For similar reasons, the analyses below treat comparative studies as multiple single-group pre–post comparisons rather than as comparative. Evidence from controlled studies is considered separately in Sect. 3.6.

### Study Quality

The results of the quality assessment are shown in Table 4. Most studies used a single-group or uncontrolled design, i.e. only one training programme was evaluated [2, 3, 12–16, 18–21, 23–25, 27–35, 37]. Therefore, these studies did not receive high quality scores for study design, confounders and blinding (sections B, C and D, respectively). Two studies [17, 36] scored slightly higher, since they used comparative designs and two of the single-group studies reported their methods more clearly therefore were able to receive higher ratings on some of the domains resulting in a higher overall rating [20, 27]. There were also substantial limitations in the reporting of sampling and recruitment (section A) and attrition (section F) in most studies. In general, most of the studies received higher scores for reliability and validity of outcome measures (section E). Therefore, generally the quality of the included studies was poor. Due to the lack of higher-quality studies, we did not exclude lower-quality evidence or attempt to weight the synthesis by quality rating.

**Table 4:** Quality appraisal

	Selection bias	Study design	Confounders	Blinding	Data collection method	Withdrawals/dropouts	Overall rating
Knapik et al. [22]^a^	Weak	Weak	Moderate	Weak	Weak	Weak	Weak
Teves et al. [32]	Weak	Weak	Weak	Weak	Moderate	Weak	Weak
Knapik et al. [23]	Moderate	Weak	Weak	Weak	Moderate	Moderate	Weak
Knapik et al. [21]	Weak	Weak	Moderate	Weak	Moderate	Weak	Weak
Bell et al. [12]	Moderate	Weak	Weak	Weak	Moderate	Weak	Weak
Hart et al. [18]	Weak	Weak	Weak	Weak	Moderate	Weak	Weak
Yanovich et al. [38]	Weak	Weak	Weak	Weak	Strong	Weak	Weak
Wood and Kruger [36]	Weak	Weak	Moderate	Weak	Moderate	Strong	Moderate
Sharp et al. [30]	Weak	Weak	Weak	Weak	Strong	Weak	Weak
Vogel et al. [33]	Weak	Weak	Weak	Weak	Strong	Weak	Weak
Evans et al. [16]	Weak	Weak	Weak	Weak	Strong	Moderate	Weak
Jetté et al. [20]	Moderate	Weak	Weak	Weak	Moderate	Moderate	Moderate
Patton et al. [27]	Moderate	Weak	Weak	Weak	Strong	Moderate	Moderate
Sharp et al. [29]	Weak	Weak	Weak	Weak	Strong	Weak	Weak
Yanovich et al. [37]	Weak	Weak	Weak	Weak	Strong	Weak	Weak
Drain et al. [15]	Weak	Weak	Weak	Weak	Moderate	Weak	Weak
Sonna et al. [31]	Weak	Weak	Weak	Weak	Strong	Weak	Weak
von Restorff [34]	Weak	Weak	Weak	Weak	Moderate	Strong	Weak
Harwood et al. [19]	Weak	Weak	Weak	Weak	Moderate	Weak	Weak
Rayson et al. [28]	Weak	Weak	Weak	Weak	Moderate	Moderate	Weak
Patterson et al. [26]	Weak	Weak	Weak	Weak	Moderate	Weak	Weak
Richmond et al. [3]	Weak	Weak	Weak	Weak	Moderate	Weak	Weak
Daniels et al. [13]	Weak	Weak	Weak	Weak	Strong	Strong	Weak
Blacker et al. [2]	Weak	Weak	Weak	Weak	Weak	Moderate	Weak
Williams et al. [35]	Weak	Weak	Weak	Weak	Moderate	Moderate	Weak
Marcinik and Hodgdon [24]	Weak	Weak	Weak	Weak	Moderate	Moderate	Weak
Mason et al. [25]	Moderate	Weak	Weak	Weak	Moderate	Weak	Weak
Gambera et al. [17]	Moderate	Strong	Moderate	Moderate	Weak	Strong	Moderate
Daniels et al. [14]	Weak	Weak	Weak	Weak	Strong	Weak	Weak

^a^Knapik et al. [22] reported as two-group, but treated as one-group for data analysis

### Aerobic Fitness

#### Maximal Oxygen Uptake

Thirteen studies measured maximum oxygen uptake ($\dot{V}{\text{O}}_{2\text{max} }$; Electronic Supplementary Materials Fig. S1), some in absolute [17, 30] and some in relative terms (per kg of body mass) [13, 14, 16, 26, 27, 30–32, 37, 38]. To provide a consistent outcome measure in this analysis, absolute values were divided by the mean body mass values reported for men and women in each study at baseline to provide these data in relative terms. The 13 studies reported data on 21 female and 19 male groups. In all 40 of these groups, $\dot{V}{\text{O}}_{2\text{max} }$ was higher after training than before; 17 of the pre–post differences were found to be significant. In all but two of 19 comparisons between men and women, pre–post differences were higher for women than for men. The median relative pre–post improvement was 7.4%; for men it was 4.0% and for women 8.2%. The median absolute pre–post improvement was 3.0 ml kg^−1^ min^−1^; for men it was 2.0 ml kg^−1^ min^−1^ and for women 3.4 ml kg^−1^ min^−1^.

Statistical comparisons between men and women were made by five of the 13 studies. One study found men had a significantly higher $\dot{V}{\text{O}}_{2\text{max} }$ than women prior to training but this was not assessed post-training [26], three studies found men had a significantly higher $\dot{V}{\text{O}}_{2\text{max} }$ than women both pre- and post-training [16, 30, 37] and one study found no significant sex by outcome interaction [27].

#### Run Time

Twelve studies measured time taken to run a certain distance as a measure of aerobic fitness (Electronic Supplementary Materials Fig. S2) [2, 3, 13, 16, 19, 21, 22, 25, 27, 28, 36, 37]. Distances varied between 1 mile (1.6 km) and 2 miles (3.2 km). Apart from differences in distance, it was unclear whether this outcome was comparable between studies as, in many cases, limited information was reported about the nature of the course (e.g. the terrain covered).

The 12 studies included data on 15 male and 15 female groups. All but one group recorded faster mean run times following training [36]; 18 of these pre–post differences were found to be significantly improved. There was a greater pre–post improvement for women than for men in all 12 studies. The median relative pre–post improvement was 9.5% overall; for men it was 5.7% and for women 10.4%. The most common distance evaluated was 1.5 miles (2.4 km, *n* = 7 studies). The median absolute pre–post improvement was 52 s overall; for men it was 31 s and for women 73 s.

Statistical comparisons between men and women were made in six of the 12 studies. Four studies found men had a significant faster run time than women both pre- and post-training [2, 16, 28, 37], one study only investigated post-training differences between men and women and found men had a significantly faster run time than women [19], and one study found no significant sex by outcome interaction [27].

#### Other Outcomes

Other outcomes reflecting aerobic and anaerobic fitness (walking, progressive/shuttle runs and power) are tabulated in Electronic Supplementary Materials Table S1. One study [36] (two male and two female groups) measured 4 km walk time, finding a 9% median pre–post difference across groups, with little difference between female and male participants. Four studies measured shuttle runs or progressive runs [19, 25, 35, 36] (six male and six female groups), finding a 5.7% median pre–post improvement; for men it was 5.4% and for women 16.1%. Improvements were observed in all groups, although statistical significance was reached only in two of the male groups and two of the female groups.

Statistical comparisons between men and women were made in only one study [19], where men ran for significantly longer on a shuttle run test than women post-training (pre-training was not reported).

Three studies measured peak power or total work using a Wingate (or similar protocol to a Wingate) cycling test ([24, 26, 37] six male and six female groups), finding a 1.7% median pre–post improvement; for men it was 0.1% and for women 3.7%. Small adverse, and insignificant, changes were observed in three of six male groups for this outcome.

Statistical comparisons between men and women were made in all three studies. One study found men had a significantly higher peak power than women prior to training but this outcome was not assessed post-training [26]. One study found men had a significantly higher peak power than women both pre- and post-training [37] and one study found no significant differences between the sexes for all outcome measures [24].

### Strength and Muscle Endurance

#### Whole Body Muscle Strength

Nine studies measured outcomes reflecting whole-body muscle strength (ten male and ten female groups) [15, 18–20, 26, 30, 32, 34, 35], of which eight provided the absolute data (Electronic Supplementary Materials Table S2; 28 cases pooled). Several of these outcomes are not strictly muscle strength outcomes but are intended to reflect specific military tasks. However, we felt these outcomes fitted better into strength rather than aerobic outcomes given their carrying and lifting nature. One study [26] measured time to complete a ‘run-dodge-jump’ assault course (two male and two female groups), and another [34], which looked at recruits being trained for military medical service, used an exercise designed to simulate carrying patients on a stretcher (one male and one female group). Several studies also measured tests of lifting heavy loads from ground level to a specified height, intended to simulate lifting tasks carried out on military operations. Across all these outcomes combined, the median pre–post improvement was 10.3%; for men it was 9.3% and for women 13.5%. Adverse differences were observed in three cases, of which one reached significance, while 18 cases significantly improved.

Statistical comparisons between men and women were made in five of the nine studies. One study found men were significantly better at the run, dodge, jump test than women prior to training but did not include post-training assessments [26]. One study found men could lift a significantly heavier weight for the incremental dynamic lifting machine at 183 cm than women [20]. Two studies found a significant sex by time interaction for lifting a box to 145–150 cm [15] and the incremental dynamic lift machine at 152 cm [30], whilst one study found no significant differences between the sexes for the incremental dynamic lift machine at 145 cm [19].

#### Whole Body Power

Three studies measured whole body power (i.e. the ability to exert a maximum muscle contraction instantly in an explosive burst of movements; Electronic Supplementary Materials Table S3; three male and three female groups, 16 cases pooled) [25, 30, 37]. Two studies measured vertical jump power [30, 37] and one study measured power in a moving lift using an Aristokin (Lode, Groningen, The Netherlands) [25]. All three studies observed some adverse effects, with a median pre–post decline of − 13.3%; for men it was − 13.3% and for women − 17.9%. A significant decline was observed in three outcomes (vertical jump height, peak power and mean power) from the same study, in both the male and female groups [30].

Statistical comparisons between men and women were made in two of the three studies. One study found no significant difference between the sexes for ground reaction force [37] and one study found a significant sex by time interaction for peak and mean power [30].

#### Muscle Endurance

Six studies measured muscle endurance (i.e. the repetition of muscle activity to exhaustion; Electronic Supplementary Materials Table S4; seven male and seven female groups, 16 cases pooled) [19, 20, 24–26, 35]. Various exercises were used for these measures, including repetition to fatigue of bicep curls, pull-ups and bench press. Across these studies, the median pre–post improvement was 19.6%; for men it was 19.6% and for women 27.2%. However, there was considerable variability in the outcomes, with no change, or a decline, in muscle endurance in five cases, and large improvements of over 50% in others. Six cases observed a significant improvement in muscle endurance [20, 24].

Statistical comparisons between men and women were made in four of the six studies. One study found men could complete significantly more pull-ups than women prior to training but this outcome measure was not assessed post-training [26]. This finding was supported by another study, albeit at post-training (they did not assess pre-training) [19]. One study found men could complete significantly more bicep curls before fatigue compared to women [20] and one study found no significant differences between the sexes for bench press and leg press until fatigue [24] (although the required weights used by males and females were set at different values).

#### Push-Ups

Six studies measured the maximum number of push-ups (press-ups) participants could perform, either in 2 min or to exhaustion [12, 21, 22, 26, 36, 37]. The six studies included data on nine male and nine female groups (Electronic Supplementary Materials Fig. S3). All but three groups recorded higher scores after training than before training. In all but three cases the pre–post improvements were higher for female participants than for men. The median relative pre–post improvement was 51.8% overall; for men it was 49.8% and for women 70.6%. This median figure conceals a wide range in the findings, with some groups showing no pre–post difference (or even an adverse difference in one case) and some showing very substantial improvements of more than 100%. Significant improvements were observed in ten of the 18 groups (five male and five female groups).

Statistical comparisons between men and women were made in three of the six studies. One study found men could complete significantly more push-ups than women prior to training but this same outcome was not assessed post-training [26] and two studies found men could complete significantly more push-ups than women both pre- and post-training [12, 37].

#### Sit-ups

Seven studies measured the number of sit-ups participants could perform. The seven studies contained data on eight male and eight female groups (Electronic Supplementary Materials Fig. S4) [12, 19, 21, 22, 25, 36, 37]. This figure does not show two studies included in the analysis here, one that used abdominal curls rather than sit-ups and so observed much larger absolute values [25], and one that measured endurance time on a progressive test, rather than the number of repetitions performed [19]. All groups recorded higher scores after training than before. In all cases the pre–post improvements were higher for female participants than for men. The median relative pre–post improvement was 47.3% overall; for men it was 35.6% and for women 53.2%. Significant improvements were observed in ten of the 18 groups (five male and five female groups).

Statistical comparisons between men and women were made by three of the seven studies. One study found men could complete significantly more sit-ups than women both pre- and post-training [12]; one study found no significant differences between men and women both pre- and post-training [37] and one study only investigated post-training differences between men and women, but also found men could complete significantly more sit-ups than women [19].

#### Upper Body Strength

Ten studies measured upper body strength, of which nine provided absolute data, using a range of specific exercises, including, among others, bench press, shoulder press and bicep curls (Electronic Supplementary Materials Table S5; 11 male and 11 female groups; 34 cases pooled) [13, 18–20, 23, 24, 26, 30, 33, 34]. Across these studies the median pre–post improvement was 8.5%; for men it was 6.9% and for women 13.0%. Adverse changes were observed in eight cases (five male and three female groups), of which two reached significance (one male and one female group), whilst 23 cases significantly improved (11 male and 12 female groups).

Statistical comparisons between men and women were made in six of the ten studies. One study found men could bench press significantly heavier weights than women, but this outcome was not assessed post-training [26]. Two studies found men could bicep curl significantly heavier weights than women pre- and post-training [20, 24]. One study found men had significantly better trunk extensor strength and upper torso strength pre- and post-training [23]. Finally, no significant differences were found between the sexes for all other studies [19, 20, 24, 30] and their outcomes (back extension, bench press, *latissimus dorsi* pulldown, shoulder arm push, shoulder press, static arm shoulder strength, elbow flexion, upper torso strength).

#### Lower Body Strength

Ten studies measured lower body strength, of which nine provided absolute data, using a range of specific exercises, including leg press, leg extensor and knee flexor strength (Electronic Supplementary Materials Table S6; 11 male and 11 female groups; 28 cases pooled) [13, 19, 23–26, 30, 32, 33, 35]. Across these studies the median pre–post improvement was 8.9%; for men it was 7.0% and for women 10.5%. Adverse changes were observed in five cases, but none reached significance, whilst significant improvements were observed in 14 cases (seven male and seven female groups).

Statistical comparisons between men and women were made in five of the ten studies. Upright pull from 38 cm was not significantly different between men and women in two studies [19, 30], but men performed significantly better than women pre-training in one study [26] (it was not assessed post-training). Leg press was not significantly different between men and women in one study pre- or post-training [24], but men were significantly better than women pre-training in another [26] (leg press was not assessed post-training). Knee extensor strength [24], leg extensor strength [23] and lower body strength [30] were all not significantly different between the sexes post-training.

#### Grip Strength

Five studies measured hand-grip strength (Electronic Supplementary Materials Table S7, six male and six female groups; 18 cases pooled) [18, 20, 26, 32, 34]. Most of these studies observed an adverse (although not significant) pre–post decline, with a median difference of − 0.5%; for men it was − 0.2% and for women − 0.7%. A significant improvement in grip strength was observed in two studies (four groups, two male and two female).

Statistical comparisons between men and women were made in two of the five studies. One study found no significant differences between the sexes for combined grip strength [20] and one study found men had a significantly stronger left and right hand grip than women prior to training but this outcome was not assessed post-training [26].

### Comparative Studies

As noted from the quality appraisal (Table 4), few (*n* = 5) studies used controlled designs.^2^ In one case [17], the only randomised trial included, the two study arms both received the same training intervention (while one also received dietary advice), meaning the randomised element is not relevant to this review. Only $\dot{V}{\text{O}}_{2\text{max} }$ was measured by this study. Significant relative improvements were observed by all four groups, but statistical analyses between the sexes were not conducted. One study [38] compared basic training received by soldiers (women and men) preparing for combat roles with less demanding training undertaken by women in non-combat military service roles. This study also only assessed $\dot{V}{\text{O}}_{2\text{max} }$ and found approximately similar relative improvements between the two groups of women over the study period. Again, statistical sex analyses were not conducted.

Three studies compared different types of training intervention. None of these studies were randomised and there was limited information on allocation, meaning there is a possibility of confounding. One study [22] compared ‘traditional’ basic combat training to a new programme, ‘Physical Readiness Training’, which incorporated a more varied range of exercises and less running, with the primary objective of reducing injuries. Similar improvements in fitness outcomes (2-mile run time, maximum push ups and sit ups in 2 min) from the two training programmes were recorded for both men and women. Following the two different training programmes, there were no significant differences between the proportion of recruits (male or female) passing the initial Army Physical Fitness Test (APFT). Significant differences between the sexes were not reported. One study [26] similarly compared a revised training programme to usual combat training. However, while a detailed breakdown of the new programme is reported in the study, no information is reported on the training received by the usual-treatment control group, so the interpretation of this study is limited. This study found significant pre–post differences for a number of outcomes (e.g. bench press, leg press, and a run, dodge, jump course) in the intervention group compared to the control group. However, with low sample sizes (female intervention *n* = 9, female control *n* = 3; male intervention *n* = 13, male control *n* = 6) these results must be interpreted with caution. Sex comparisons were made for the fitness outcomes prior to training (all outcomes were significantly better in men compared to women) but were not reported post-training. Finally, one study [36] compared a revised ‘cyclic-progressive’ training programme to usual basic combat training (BCT), with the revisions including more jogging, upper body and abdominal exercises, and less warm-up and games (approximately the opposite to the findings of Knapik et al. [22]). This study found significantly greater improvements for intervention than control participants in both strength and aerobic fitness for both men and women. Again, sex comparisons were not reported.

## Discussion

Here we present the first systematic review of the literature investigating the changes in physical performance over a period of military training in men and women. It was previously unclear whether sex differences exist in the adaptation to military training and, therefore, whether sex-specific training should be employed to optimise training adaptations. Despite all retrieved studies containing both male and female groups undergoing the same training, few studies statistically evaluated study outcomes by sex. In studies where sex differences were statistically evaluated, there were typically no differences in the physical performance adaptations to training between sexes. However, sex differences were evident at pre- and/or post-training time-points across a range of performance components. Aerobic fitness and muscle strength were most consistently increased across all study groups following military training, with more varied, inconsistent results in components of fitness/performance including muscle endurance, push-ups, sit-ups and lower body muscle strength. This systematic review provides a novel and comprehensive insight into sex differences in the performance response to military training.

Sex differences in the physical performance response to military training were statistically evaluated in 51% of studies. Statistical analyses varied among studies with some studies assessing the sex by outcome/time interaction, and other studies only evaluating pre- or post-training differences. Sex differences were observed in 63% of studies evaluating sex differences, although the majority of these studies (87%) demonstrated significant sex differences pre- and post-training, or pre-/post-training only, rather than a sex by outcome/time interaction. These data suggest that the physical performance response in men and women undergoing military training is similar (i.e. both men and women will improve following a training programme), yet highlight clear performance differences between the sexes prior to training that are not negated with military training (i.e. men perform better on the pre-training physical tests and remain better post-training when compared to women).

The lack of any apparent divergent responses for men and women to military physical training is promising in that existent training practices, despite often being inherited from typically male-orientated training environments, are not limiting for women. However, we are also unable to say whether military training is currently in its most effective form for both men and women, acknowledging the impact of competing demands/constraints inherent within BMT, and the fact that training is largely designed for expediency, large numbers and limited resources [39]. Given that the physical performance of women following military training is not, on average, at an equivalent level to that of men, specific physical training programmes may need to be developed and evaluated for women, particularly if women are to operate successfully in physically arduous GCC roles. The training gains of ~ 10% across a number of outcomes documented in our systematic review and other studies [2, 3] are smaller than can be achieved in women with specific, progressive, periodised training [39–41], and suggest that alternative training programmes may need to be employed to support women in passing the physical employment standards [42] of GCC roles and sustaining a successful GCC career. Future work should consider whether current military training is most effective in its current form for both men and women, or whether alternative training programmes would be more effective in developing physical performance across the range of performance components.

Due to the physical demands of GCC training and employment, it is necessary for military training to effectively develop a range of physical performance attributes, including aerobic endurance, anaerobic endurance, muscle strength, muscle power and mobility. Our data demonstrate that aerobic endurance and muscle strength (whole body and upper body) performance were improved most consistently across studies in our review, with the vast majority of studies showing significant improvements in these metrics. Although sit-up and press-up performance tests had the greatest median improvement of all the performance outcomes, significant improvements were only measured in 56% of cases. Whole body power appeared to be adversely affected by military training with a negative median change, although only one study demonstrated a significant decrement in performance, with all other studies showing no significant change. Muscle endurance and lower body muscle strength were significantly improved in 38% and 50% of cases, respectively. These data suggest that military training leads to gains in some, but not all, components of fitness.

Improvements in aerobic endurance over the course of BMT have been demonstrated in a number of studies [2, 3]. Typically, military training involves a high volume of running or locomotion on foot and thus it is not surprising that aerobic fitness is developed during this period. Moreover, aerobic fitness is a key component of load carriage performance [8], an essential military activity performed frequently in BMT. Muscle strength is also considered a key performance attribute for military personnel, with 88% of military tasks involving lifting and carrying of some nature [28] and resistance training being important for load carriage performance [8]. However, performing both endurance and resistance exercise concurrently, as is typical of military training, can result in an interference effect [43], whereby the adaptations that would arise from training each exercise type in isolation are attenuated. The improvements in aerobic endurance from the physical training programmes, combined with the fact that running endurance training results in greater lower body strength interference than other modes of endurance training [44], may explain our findings of improved upper body strength, but typically not lower body strength. Considering the requirement for GCC soldiers to lift and carry heavy loads, often over long distances, combined with aerobic endurance and strength training being essential components of load carriage performance [8], developing both whole body strength/power and aerobic fitness will be critical for success in GCC roles. Although men typically outperform women on physical tests, British Army data demonstrate an overlap in physical performance between men and women whereby the highest performing women outperform the lowest performing men. The greatest overlap is observed in the 1.5-mile endurance run, with the least overlap in the Powerbag lift strength test, suggesting that strength may be the fitness component requiring greatest attention for women. Targeted efforts to effectively physically develop trainees and serving military personnel in a multi-exercise training environment need to be prioritised, particularly for the female GCC soldier who will typically display lower physical capability than her male counterpart.

Sex comparisons within each performance component, in general, are largely reflected by the overall sex comparisons discussed previously. Often sex comparisons were not made and in instances where statistical comparisons of data between sexes were evaluated, the predominant finding tended to represent pre-/post-training differences rather than any interaction effect. These data suggest that attention needs to be afforded to both men and women, optimising delivery of physical training to achieve the most effective gains in all components of physical performance of relevance to the military.

In summary, given that enhancing performance of a specific physical capability is not the primary aim of BMT, with little recovery time to effectively adapt to physical training and the potential for interference effects from different training modalities, we are unable to conclusively answer the question of whether men and women respond differently to targeted physical training. However, the large participant numbers, within-subject pre–post design, and the ‘real-life’ application of the included studies does allow us to conclude that the physical performance of men and women in a number of attributes is improved over the course of BMT. Moreover, the relative gains in these performance attributes are not compromised in women compared to men, suggesting that both sexes have the capacity to effectively improve their physical performance during BMT. Understanding the impact of training with different exercise modes on overall physical adaptation, including mechanistic differences between men and women, will be important in our understanding of whether men and women need to be trained differently to optimise the response to physical training in both sexes. Future work reviewing the training literature outside of the military environment may provide a greater understanding of the mechanisms that underpin sex differences in the response to training programmes, facilitating the design of effective training programmes for military personnel.

### Limitations in the Evidence Base

The major gap in the evidence base identified by this review is the lack of controlled prospective studies (i.e. studies that have a control group completing BMT and an intervention group completing a new training programme), ideally randomised trials, of training interventions in military populations that statistically compare sex differences. This review located very few studies using controlled designs, with relevant comparisons. Instead, studies were typically a single intervention with a pre/post design, and these studies were generally rated as low-quality evidence from our quality appraisal. There is a substantial body of evidence reporting the response of male and female personnel to training, particularly initial military training. However, these studies can be treated as studies of effectiveness only to a limited extent (and, indeed, in many cases do not seem to be conceived as such by their authors): the absence of comparison groups limits the internal validity of the findings and makes it difficult to synthesise the results quantitatively. Higher quality evidence would be obtained if studies were designed to specifically investigate sex differences in the response to physical training. This would offer greater insight into whether men and women respond differently to physical training, and such studies should be prioritised in future if we are to develop effective physical training programmes for military personnel.

Given that most studies were conducted during BMT, the time frame of sampling matched the lengths of these initial training programmes (approximately 3 months in most cases). The lack of evidence on longer-term outcomes may be of concern for two reasons: we are unable to determine firstly, whether these initial training improvements are maintained over time in both men and women, and secondly, whether longer training programmes result in continued fitness improvements in both men and women.

Apart from the use of uncontrolled single-group designs, the studies have several other methodological limitations. Selection bias may be pertinent since sampling and recruitment information was limited across all studies. Limitations in reporting of the methods used for each fitness test preclude conducting indirect comparisons across studies (since they would be heavily confounded). In addition, many studies presented limited information on the content of the physical training undertaken. Finally, the results do not support conclusions about the relative effectiveness of different training regimens or environments.

A further limitation of the evidence was that most studies analysed those who completed, and excluded those who dropped out of, training. In many cases attrition rates were substantial and the reasons were not always clear. However, the majority of the attrition seems to reflect participants either being injured or being discharged from the military for other reasons, rather than simple loss to follow-up. The pre–post results extracted and analysed in this review effectively ignore these participants. From a practical viewpoint, the impact of the training intervention on participants who do not complete training is arguably of secondary importance. Nonetheless, the limited data on dropouts in most studies, and the absence of controlled studies using intent-to-treat analysis, means that it is unclear what impact attrition may have had on the reported changes in physical performance.

Although some studies attempted to evaluate changes in military-specific task performance using outcome measures more aligned to the physical demands of military performance [26, 34, 35], most studies used standard tests of physical performance. The relevance of standardised outcome measures for practice is not always clear, particularly in the highly variable and challenging environment of the battlefield. These limitations regarding applicability warrant consideration, although the benefit of valid, repeatable and sensitive standardised measures should not be overlooked, particularly when the objective is to compare performance between the sexes across different physical training programmes.

### Limitations of the Review

This review was based on robust systematic review methodology, including extensive and highly sensitive searches, screening using a priori criteria, and transparent processes for data extraction and synthesis. The result of these methods is a comprehensive evidence base that has been produced with minimal bias in the selection of studies and findings. However, these methods are not without their limitations.

Based on 96.4% agreement, 90% of the studies in this review were single screened by CC, JVC and TL. Whilst single screening is a potential technical limitation to this review, the review team are experienced systematic reviewers, who also conducted extensive supplementary searches.

The need to draw clear boundaries regarding inclusion criteria resulted in material that may have initially seemed relevant being excluded. Typical examples of excluded studies were those that used different measures at baseline and post-test, or for men and women, and studies comparing different samples (i.e. not the same individuals) at baseline and post-test. The review aims were to include only prospective studies, but this criterion was not applied strictly since the reporting of studies often did not allow clear determination of whether studies were prospective or not. Nonetheless, studies were excluded where it was clearly stated that a retrospective design was used. Purely observational studies, i.e. studies that did not include an intervention (or only compared outcomes between men and women at a single time point), were excluded. Such studies may have been contextually relevant, but did not enable assessment of the impact of physical training.

The limitations of the evidence base (Sect. 4.1) precluded a full meta-analysis, which could have produced pooled effect sizes for the outcomes evaluated. Instead, full outcome data have been presented (where appropriate) and unstandardised, unweighted median pre–post differences to characterise the overall findings were used. While this approach also has some limitations, and the data presented should not be confused with a full meta-analysis, presentation of the data in this manner provides an indication of the magnitude of the changes observed in the studies. It should also be noted that comparing the pre- and post-training mean values for the whole group, and expressing this difference as a change score, may often give very different results to taking the mean of the change scores for each individual.

## Conclusions

We present a systematic review of performance responses to physical training in military men and women. Typically, there were no sex differences in the physical performance adaptation to military training. Changes in aerobic endurance and muscle strength (whole body and upper body) outcomes were more consistently observed across study groups than changes in muscle power, lower body muscle strength and muscle endurance. Outcome measures of these physical performance parameters were largely not military-specific activities and thus may have not adequately represented changes in military-specific physical performance. Moreover, many of the included studies were not of a prospective, randomised, controlled trial design, but rather an evaluation of changes in physical performance over the course of BMT. Future work should focus on evaluating sex differences in response to physical training designed to improve a specific physical capability, and to understand the mechanisms underpinning adaptation to physical training in both sexes.

## Electronic supplementary material

Below is the link to the electronic supplementary material.

Supplementary material 1 (DOCX 301 kb)

Supplementary material 2 (DOCX 246 kb)

## Footnotes

1 For the purpose of this review, a systematic review was defined as one that has: a focused research question; explicit search criteria that are available to review, either in the document or on application; explicit inclusion/exclusion criteria, defining the population(s), intervention(s), comparator(s), and outcome(s) of interest; a critical appraisal of included studies, including consideration of internal and external validity of the research; and a synthesis of the included evidence, whether narrative or quantitative.

2 One further study by Knapik et al. [21] also used a controlled design, but the data reported do not allow interpretation in terms of a comparison of effectiveness of the arms.
