# Supplementary material for: Sex-Specific Changes in Physical Performance Following Military Training: A Systematic Review
Source: Sports Med. 2018 Sep 19;48(11):2623–40. doi: 10.1007/s40279-018-0983-4 (PMC6182553; doi:10.1007/s40279-018-0983-4)
Supplement: Supplementary file 1 — Supplementary material 1 (DOCX 301 kb) [file 40279_2018_983_MOESM1_ESM.docx]

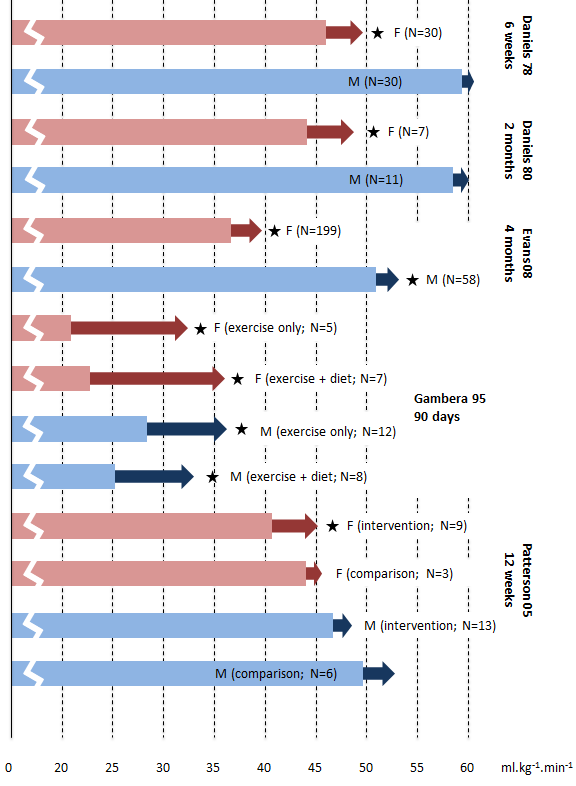

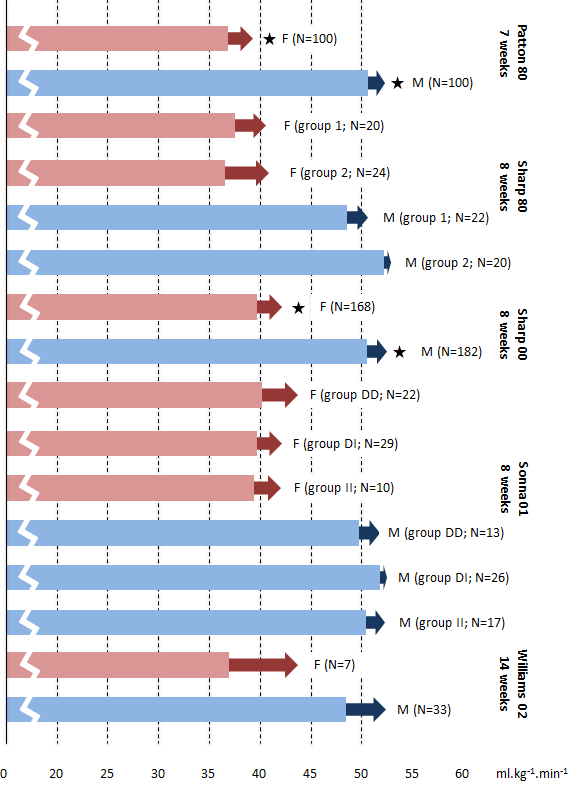

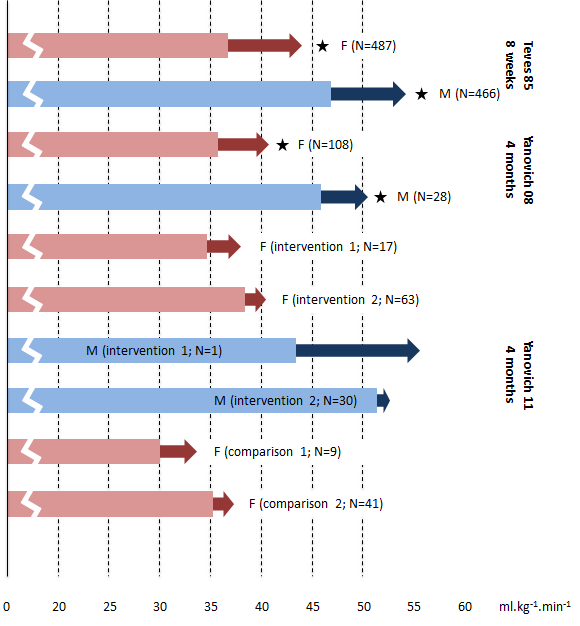


Electronic Supplementary Material Figure S1 V̇O2 max pre to post training

**Abbreviations:** F, female; M, male; N, number

Key: Star represents a significant change; arrow represents the direction and magnitude of change from pre to post training.


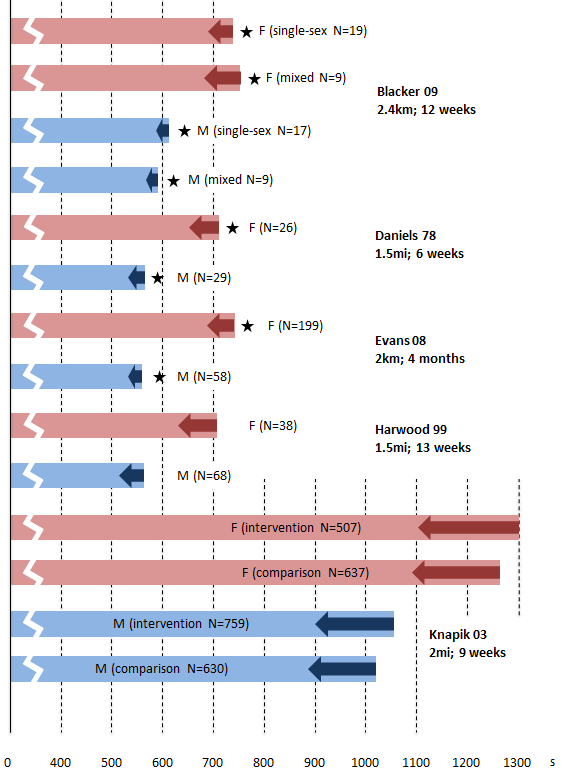

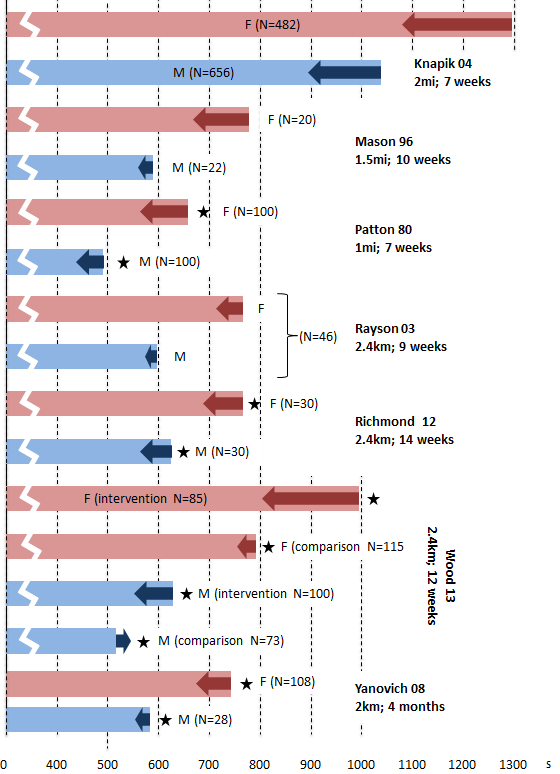


Electronic Supplementary Material Figure S2 Run Time pre to post training

**Abbreviations:** F, female; M, male; N, number; s, seconds.

Key: Star represents a significant change; arrow represents the direction and magnitude of change from pre to post training.


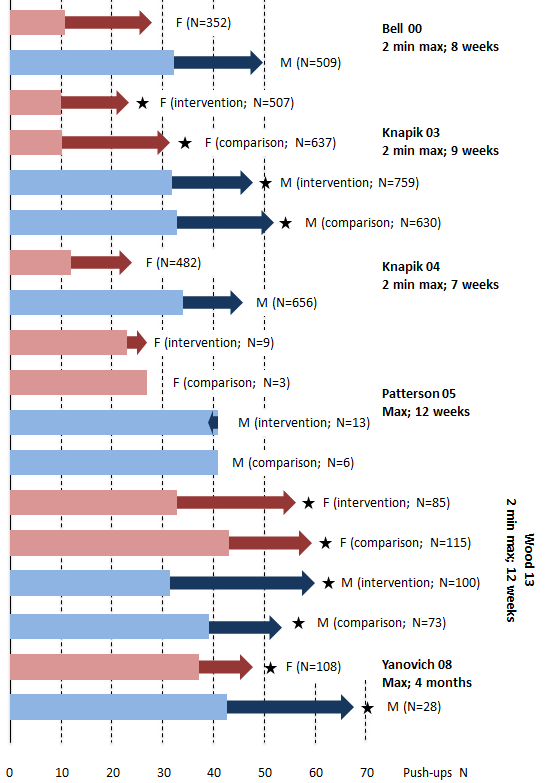


Electronic Supplementary Material Figure S3 Push Ups pre to post training

**Abbreviations:** F, female; M, male; N, number

Key: Star represents a significant change; arrow represents the direction and magnitude of change from pre to post training.


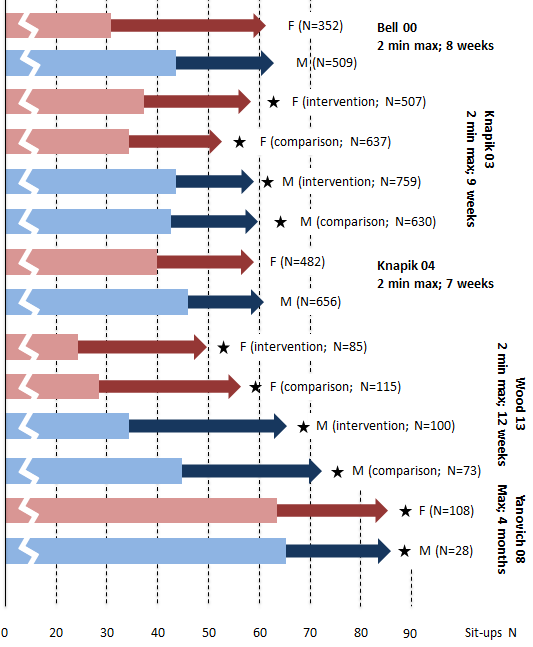


Electronic Supplementary Material Figure S4 Sit-Ups pre to post training

**Abbreviations:** F, female; M, male; N, number

Key: Star represents a significant change; arrow represents the direction and magnitude of change from pre to post training.

Electronic Supplementary Material Table S1 Aerobic/Anaerobic measures pre to post training

| Activity (units) | | Study | Participants | (n) | Pre mean (sd) | Post mean (sd) | % Change | |  |
| --- | --- | --- | --- | --- | --- | --- | --- | --- | --- |
| Walking | | | | | | | |  |  |
|  | 4km  Walk Time (min) | Wood 2013 | Female int | 85 | 35 (1.8) | 31.8 (2.1) | -9.1 | |  |
|  |  |  | Female comp | 115 | 33.8 (2.3) | 30.8 (1.9) | -8.9 | |  |
|  |  |  | Male int | 100 | 30.1 (1.5) | 27.4 (1.7) | -9.0 | |  |
|  |  |  | Male comp | 73 | 29.4 (3.0) | 27 (2.4) | -8.2 | |  |
| Running | | | | | | | |  | |
|  | Shuttle Runs (min)^b^ | Wood 2013 | Female int | 85 | 67.5 (8.1) | 65.1 (6.0) | -3.6 | |  |
|  |  |  | Female comp | 115 | 63.1 (6.7) | 60.4 (6.4 ) | -4.3 | |  |
|  |  |  | Male int | 100 | 55.4 (3.6) | 53.1 (3.1) | -4.2 | |  |
|  |  |  | Male comp | 73 | 51.2 (4.1) | 48.2 (4.2) | -5.9 | |  |
|  | Progressive Run (s)^c^ | Mason 1996 | Female | 20 | 309.4 (NR) | 381.7 (NR) | 23.4 | |  |
|  |  |  | Male | 22 | 512.2 (NR) | 530.9 (NR) | 3.7 | |  |
|  |  | Harwood 1999 ^a^ | Female | 38 | 515 (71) | 598 (65) | 16.1 | |  |
|  |  |  | Male | 68 | 720 (75) | 759 (70) | 5.4 | |  |
|  |  | Williams 2002^a^ | Female | 7 | 371 (41) | 488 (25) | 31.5 | |  |
|  |  |  | Male | 33 | 570 (70) | 639 (65) | 12.1 | |  |
|  | Loaded March with 15kg (s) | Williams 2002 | Female | 8 | 1469 (87) | 1361 (109) | -7.4 | |  |
|  |  |  | Male | 12 | 1153 (87) | 1035 (67) | -10.2 | |  |
| Cycling | | | | | | | |  | |
|  | Peak Power (W/kg)^d^ | Yanovich 2008^a^ | Female | 108 | 4.9 (0.9) | 4.9 (0.8) | 0.8 | |  |
|  |  |  | Male | 28 | 6.8 (0.9) | 6.6 (2.3) | -2.9 | |  |
|  |  | Patterson 2005^a^ | Female int | 9 | 7.59 (0.87) | 7.97 (1.22) | 5.0 | |  |
|  |  |  | Female comp | 3 | 8.30 (0.72) | 8.50 (0.68) | 2.4 | |  |
|  |  |  | Male int | 13 | 9.67 (1.33) | 9.80 (1.75) | 1.3 | |  |
|  |  |  | Male comp | 6 | 9.37 (0.67) | 9.33 (1.07) | -0.4 | |  |
|  | 30s Work (kJ) | Patterson 2005^a^ | Female int | 9 | 11.5 (1.1) | 12.4 (1.6) | 7.8 | |  |
|  |  |  | Female comp | 3 | 11.8 (1.2) | 12.6 (2.8) | 6.8 | |  |
|  |  |  | Male int | 13 | 17.5 (1.7) | 17.6 (2.5) | 0.6 | |  |
|  |  |  | Male comp | 6 | 17.4 (1.2) | 17.3 (1.0) | -0.6 | |  |
|  | Max Work Capacity (kpm)^e^ | Marcinik 1985 | Female | 9 | 1089.3 (249.2) | Increased by 2% | 2 | |  |
|  |  |  | Male | 29 | 1661.1 (276.5) | Increased by 3% | 3 | |  |

**Notes:** a, significant difference between sexes; b, running 22m 10 times without breaks; c, multi-stage fitness test; d, Wingate anaerobic test; e,cycle test to exhaustion with progressive increase (228kpm/min) of workload, max workload before fatigue reached recorded
**Abbreviations:** comp, comparison; int, intervention; n, number; nr, not reported; sd, standard deviation;
**Colour Key:** Dark Green, significantly improved; Dark Red, significantly declined; Light green, improved but not significantly or significance not reported; light red, declined but not significantly or significance not reported

Electronic Supplementary Material Table S2 Whole body muscular strength pre to post training

| Activity (units) | Study | Participants | (n) | Pre  mean (sd) | Post  mean (sd) | % Change |
| --- | --- | --- | --- | --- | --- | --- |
| IDLM to 145cm (kg)^b,j^ | Harwood 1999 | Female | 38 | 42.9 (5.6) | 40.8 (4.1) | -4.9 |
|  |  | Male | 68 | 70.4 (9.7) | 69.8 (10.1) | -0.9 |
|  | Williams 2002 | Female | 8 | 34.1 (4.4) | 39.4 (5.6) | 15.5 |
|  |  | Male | 38 | 62.6 (12.9) | 71.4 (11.5) | 14.1 |
| IDLM to 152cm (kg)^b^ | Teves 1985^k^ | Female | 487 | 30.3 (5.3) | 34.4 (5.6) | 13.5 |
|  |  | Male | 465 | 60.9 (10.9) | 65.5 (10.7) | 7.6 |
|  | Hart 1985 | Female | 278 | 13.2 (NR) | P<0.001 | sig change |
|  |  | Male | 309 | 4.5 (NR) | P<0.001 | sig change |
|  | Sharp 2000 ^d^ | Female | 99 | 40.7 (10.6) | 42.1 (9.6) | 3.4 |
|  |  | Male | 99 | 76.5 (14.8) | 73.2 (13.6) | -4.3 |
| IDLM to 183cm (kg)^b^ | Teves 1985 ^k^ | Female | 487 | 26.2 (4.7) | 30.4 (5.1) | 16.0 |
|  |  | Male | 465 | 56.9 (10.7) | 62.2 (10.9) | 9.3 |
|  | Hart 1985 | Female | 278 | 10.7 (NR) | P<0.001 | sig change |
|  |  | Male | 309 | 7.1 (NR) | P<0.001 | sig change |
|  | Jetté 1989^a^ | Female | 68 | 24.4 (4.1) | 26.6 (3.7) | 9.0 |
|  |  | Male | 76 | 39.8 (8) | 44.6 (6.3) | 12.1 |
| Box Lift to 145-150cm (kg)^c^ | Drain 2015^a^ | Female | 20 | 21.9 (3.2) | 24.9 (3.6) | 13.7 |
|  |  | Male | 154 | 44.3 (9.7) | 46.5 (8.8) | 5.0 |
|  | Williams 2002 | Female | 8 | 28.1 (5.3 | 31.8 (7.8) | 13.2 |
|  |  | Male | 11 | 71.3 (13.2) | 80.0 (12.0) | 12.2 |
| Carrying Simulated Patient of 60kg (m/s)^d^ | von Restorff 2000 | Female | 62^e^ | 1.67 (0.24) | 1.73 (0.32) | 3.6 |
|  |  | Male | 48^f^ | 1.95 (0.47) | 2.27 (0.57) | 16.4 |
| Run, Dodge Jump (s)^g^ | Patterson 2005^a^ | Female int | 6 | 90.5 (25.5) | 66.8 (9.8) | -26.2 |
|  |  | Female comp | 2 | 72.2 (2.7) | 67 (12.6) | -7.2 |
|  |  | Male int | 12 | 52.4 (5.8) | 48.7 (5.8) | -7.1 |
|  |  | Male comp | 6 | 53.5 (7.6) | 49.1 (3.7) | -8.2 |
| Lift from Squat Position (N)^h^ | von Restorff 2000 | Female | 62 | 898.06 (151.94) | 1025.77 (165.67) | 14.2 |
|  |  | Male | 48 | 1456.17 (234.06) | 1612.93 (266.66) | 10.8 |
| Lift from Standing Position (N)^i^ | von Restorff 2000 | Female | 62 | 486.4 (75.3) | 586.6 (199.9) | 20.6 |
|  |  | Male | 48 | 952.39 (182.03) | 1046.13 (219.14) | 9.8 |

Notes: a, significantly different between sexes; b, lifting of a load to the height specified, the load started between 18.1 – 27.2 kg and increased by 2.3, 2.5, 4.5 or 5kg depending on protocol, until fatigue, max load lifted was recorded; c, 145cm for Williams, 150cm for Drain; increase in weight of box by 2.5kg, 4kg or 5kg until failure; d, time taken to carry the simulated patient around an indoor volleyball court (55m), data also available for 70, 80 and 90kg persons; e, post n=55; f, post n=36; h, time taken to complete the run, dodge jump course; i, no detail on how high the lift was to; j, up to 32, 2.5kg could be added from a starting point of 20kg; k, load range between 18.1-90.9kg
Abbreviations: comp, comparison; IDLM, Incremental Dynamic Lifting Machine; int, intervention; n, number; sd, standard deviation; sig, significant
Colour Key: Dark Green, significantly improved; Dark Red, significantly declined; Light green, improved but not significantly or significance not reported; light red, declined but not significantly or significance not reported

Electronic Supplementary Material Table S3 Whole body power pre to post training

| Activity | Study | Participants | (n) | Pre  mean (sd) | Post  mean (sd) | % Change |
| --- | --- | --- | --- | --- | --- | --- |
| Ground Reaction Force Plate  (N^.^kg^-1^)^b^ | Yanovich 2008 | Female | 108 | 46.1 (9.2) | 42.7 (7.2) | -7.4 |
|  |  | Male | 28 | 49.9 (12.9) | 44.6 (8.3) | -10.6 |
| Vertical Jump Height (cm) | Sharp 2000 | Female | 97 | 33.2 (5.2) | 32.8 (5.7) | -1.2 |
|  |  | Male | 99 | 51.1 (8.2) | 49.7 (8.1) | -2.7 |
| Peak Power (W)^c^ | Sharp 2000^a^ | Female | 97 | 2512 (566) | 2496 (554) | -0.6 |
|  |  | Male | 99 | 4191 (685) | 4050 (667) | -3.4 |
| Mean Power (W)^c^ | Sharp 2000^a^ | Female | 97 | 766 (290) | 765 (276) | -0.1 |
|  |  | Male | 99 | 1511 (341) | 1446 (321) | -4.3 |
| Lift Mean Power (J.s.^-1^)^d^ | Mason 1996 | Female | 20 | 106.6 (NR) | 72.6 (NR) | -31.9 |
|  |  | Male | 22 | 191.8 (NR) | 137.6 (NR) | -28.3 |
| Lift Max Power (J.s.^-1^)^d^ | Mason 1996 | Female | 20 | 225.1 (NR) | 161 (NR) | -28.5 |
|  |  | Male | 22 | 362.6 (NR) | 304.8 (NR) | -15.9 |
| Lift Total Work (J)^d^ | Mason 1996 | Female | 20 | 279.9 (NR) | 136.6 (NR) | -51.2 |
|  |  | Male | 22 | 503.1 (NR) | 261.6 (NR) | -48.0 |
| Lift Max Force (N)^d^ | Mason 1996 | Female | 20 | 562.5 (NR) | 402.9 (NR) | -28.4 |
|  |  | Male | 22 | 906.3 (NR) | 762.1 (NR) | -15.9 |

**Notes:** a, significant differences between females and males at pre-training time point; b, ground reaction force plate was used to assess lower extremity force and power, single and double leg jumps were performed to calculate force per kg; c, calculated from jump height; d, no details on how these measures were assessed
**Abbreviations:** comp, comparison; int, intervention; n, number; nr, not reported; sd, standard deviation;
**Colour Key:** Dark Green, significantly improved; Dark Red, significantly declined; Light green, improved but not significantly or significance not reported; light red, declined but not significantly or significance not reported

Electronic Supplementary Material Table S4 Muscular Endurance, all activities to exhaustion or within time limit (reps) pre to post training

|  | **Activity** | **Study** | **Participants** | **(n)** | **Pre  mean (sd)** | **Post  mean (sd)** | **% Change** |
| --- | --- | --- | --- | --- | --- | --- | --- |
| Upper Body | | | | | | | |
|  | Bicep Curls^b^ | Jetté 1989^a^ | Female | 68 | 5.8 (3.8) | 9.1 (4.3) | 56.9 |
|  |  |  | Male | 76 | 8.4 (4.7) | 10.3 (4.3) | 22.6 |
|  | Pull Ups | Harwood 1999^a^ | Female | 38 | 0.7 (1.7) | 1.3 (1.8) | 85.7 |
|  |  |  | Male | 68 | 8.3 (4.1) | 10.6 (3.7) | 27.7 |
|  |  | Patterson 2005^a^ | Female int | 9 | 1 (1) | 1 (2) | 0.0 |
|  |  |  | Female comp | 3 | 2 (3) | 2 (3) | 0.0 |
|  |  |  | Male int | 13 | 11 (6) | 10 (4) | -9.1 |
|  |  |  | Male comp | 6 | 11 (6) | 10 (5) | -9.1 |
|  |  | Mason 1996^c^ | Female | 20 | 0.12 (NR) | 0.11 (NR) | -8.3 |
|  |  |  | Male | 22 | 6.4 (NR) | 7.1 (NR) | 10.9 |
|  | Bench Press^d^ | Marcinik 1985 | Female | 9 | 14 (7) | +64 % | 64 |
|  |  |  | Male | 29 | 16.9 (4.5) | +40 % | 40 |
| Lower Body | | | | | | |  |
|  | Leg Press^c^ | Marcinik 1985 | Female | 9 | 36.7 (25.2) | +44 % | 44 |
|  |  |  | Male | 29 | 39.3 (13.4) | +29 % | 29 |
| Whole Body | | | | | | | |
|  | Repetitive Lift and Carry for 10m^e^ | Williams 2002 | Female | 8 | 43.9 (3.9) | 48.4 (4.0) | 10.3 |
|  |  |  | Male | 12 | 54.7 (2.2) | 63.8 (5.5) | 16.6 |

**Notes:** a, significant difference between sexes; b, females curled a weight of 18.1kg and males a weight of 27.2 kg; c, reported as heaves; d, at 60% of 1 rep max; e, max number of repetitive lift and carry within 10 minutes
**Abbreviations:** comp, comparison; int, intervention; n, number; nr, not reported; sd, standard deviation;
**Colour Key:** Dark Green, significantly improved; Dark Red, significantly declined; Light green, improved but not significantly or significance not reported; light red, declined but not significantly or significance not reported

Electronic Supplementary Material Table S5 Upper body strength pre to post training

| Activity (units) | Study Name | Participants | (n) | Pre mean (sd) | Post mean (sd) | % Change |
| --- | --- | --- | --- | --- | --- | --- |
| Bench Press (kg)^b^ | Patterson 2005 ^a^ | Female int | 9 | 52.8 (11.8) | 60.6 (16.2) | 14.8 |
|  |  | Female comp | 3 | 56.1 (15.6) | 56.5 (22) | 0.7 |
|  |  | Male int | 13 | 117.1 (26.1) | 133.6 (28.1) | 14.1 |
|  |  | Male comp | 6 | 123.3 (26.4) | 121.3 (43.0) | -1.6 |
|  | Marcinik 1985 | Female | 9 | 28.9 (5.8) | +14 % | 14.0 |
|  |  | Male | 29 | 53.3 (9.3) | +10 % | 10.0 |
| Shoulder Press (Marcinik, kg;  von Restorff, N) | Marcinik 1985^b^ | Female | 9 | 25.5 (2.4) | +15 % | 15.0 |
|  |  | Male | 29 | 44.3 (7.4) | +15 % | 15.0 |
|  | von Restorff 2000^d^ | Female | 62 | 922.5 (247.8) | 1081.4 (271.7) | 17.2 |
|  |  | Male | 48 | 1533.0 (378.2) | 1612.9 (267.7) | 5.2 |
| Lat Pulldown (kg)^b^ | Marcinik 1985 | Female | 9 | 28.9 (5.4) | +12 % | 12.0 |
|  |  | Male | 29 | 56.6 (6.9) | +16 % | 16.0 |
| Upper Torso Strength (kg) | Knapik 1980^a,d^ | Female | 359 | 55.3 (11.8) | 61 (9.6) | 10.3 |
|  |  | Male | 733 | 97.8 (18.2) | 102.1 (16.2) | 4.4 |
|  | Daniels 1980^d^ | Female | 7 | 66.8 (1.9) | 70.5 (1.9) | 5.5 |
|  |  | Male | 11 | 106.6 (4.7) | 113.9 (3.7) | 6.8 |
|  | Sharp 2000^d^ | Female | 99 | 65.6 (11.4) | 67.3 (11.3) | 2.6 |
|  |  | Male | 99 | 113.7 (17.3) | 113.2 (16.2) | -0.4 |
| Elbow Flexion/  Bicep Curl (kg) | Vogel 1977^d^ | Female | 159 | No sig change |  |  |
|  |  | Male | 186 | No sig change |  |  |
|  | Jetté 1989^a,d^ | Female | 68 | 23.1 (4.3) | 26.3 (5.6) | 13.9 |
|  |  | Male | 76 | 44.9 (9) | 43.8 (8.6) | -2.4 |
|  | Marcinik 1985 ^a,c^ | Female | 9 | 14.2 (1.6) | +24 % | 24.0 |
|  |  | Male | 29 | 31 (4.8) | +13 % | 13.0 |
| Elbow Extension | Vogel 1977^d^ | Female | 159 | No sig change |  |  |
|  |  | Male | 186 | No sig change |  |  |
| Shoulder Arm Push (mm) | Jetté 1989^d^ | Female | 68 | 32.6 (5.8) | 37.3 (6) | 14.4 |
|  |  | Male | 76 | 55.2 (9.5) | 59.7 (10.3) | 8.2 |
| Upper Body Exertion | Hart 1985^d^ | Female | 278 | 8% increase, P<0.0001 | | 8 |
|  |  | Male | 309 | 1.6 % increase, P<0.05 | | 1.6 |
| Static Arm/Shoulder Strength (kg) | Marcinik 1985^e^ | Female | 9 | 37.2 (7.9) | +16 % | 16.0 |
|  |  | Male | 29 | 63 (9) | +12 % | 12.0 |
| Static Elbow Flexion (kg)^c^ | Marcinik 1985^d^ | Female | 9 | 28.06 (6.4) | -10 % | -10.0 |
|  |  | Male | 29 | 43 (8.5) | +7 % | 7.0 |
| Trunk Extensor Strength (kg) | Knapik 1980^a,d^ | Female | 360 | 47.6 (12.7) | 56.6 (10.6) | 18.9 |
|  |  | Male | 750 | 72.6 (18.2) | 79 (16.5) | 8.8 |
|  | Daniels 1980^d^ | Female | 7 | 59.2 (2.8) | 55 (2.5) | -7.1 |
|  |  | Male | 11 | 87.1 (3.5) | 79.7 (3.1) | -8.5 |
| Back Extension (kg) | Harwood 1999^d^ | Female | 38 | 79.8 (9.9) | 78.1 (10.4) | -2.1 |
|  |  | Male | 68 | 125 (17) | 118 (20.3) | -5.6 |

**Notes:** a, significant differences between females and males; b, measured as 1 rep max; c, reported as 2-armlift; d, measured by dynamometer; e, reported as 1-arm pull, subjects pulled a handle while bracing their other hand on a pole;
**Abbreviations:** comp, comparison; int, intervention; n, number; nr, not reported; sd, standard deviation;
**Colour Key:** Dark Green, significantly improved; Dark Red, significantly declined; Light green, improved but not significantly or significance not reported; light red, declined but not significantly or significance not reported

Electronic Supplementary Material Table S6 Lower body strength pre to post training

| Activity | Study | Participants | (n) | Pre | Post | % Change |  |
| --- | --- | --- | --- | --- | --- | --- | --- |
| Lower Body Strength (kg) | Sharp 2000^d^ | Female | 88 | 96.7 (24.7) | 103.3 (25.6) | 6.8 |  |
|  |  | Male | 85 | 160.5 (42.6) | 162.3 (39.9) | 1.1 |  |
| Leg Extensor Strength (kg) | Knapik 1980^d^ | Female | 348 | 93.4 (30) | 106.6 (31.1) | 14.1 |  |
|  |  | Male | 733 | 143.2 (38.4) | 158.2 (41.1) | 10.5 |  |
|  | Daniels 1980^e^ | Female | 7 | 124.2 (6.7) | 134.5 (9.4) | 8.3 |  |
|  |  | Male | 11 | 181 (9.7) | 193.6 (8.4) | 7.0 |  |
| Leg Press (kg)^b^ | Patterson 2005^b^ | Female int | 9 | 101.1 (15.5) | 115.3 (12.9) | 14.0 |  |
|  |  | Female comp | 3 | 105.2 (8.9) | 109.4 (13.3) | 4.0 |  |
|  |  | Male int | 13 | 139.8 (25.6) | 162.8 (24.8) | 16.5 |  |
|  |  | Male comp | 6 | 142.1 (18.36) | 150.2 (14.6) | 5.7 |  |
|  | Marcinik 1985 | Female | 9 | Unreadable | +16% | 16.0 |  |
|  |  | Male | 29 | 163.5 (23.6) | +10% | 10.0 |  |
| Knee Extensor Strength (kg) | Marcinik 1985^c^ | Female | 9 | Unreadable | +31% | 31.0 |  |
|  |  | Male | 29 | 47.9 (10.2) | +25% | 25.0 |  |
|  | Vogel 1977^d^ | Female | 159 | No sig change | |  | |
|  |  | Male | 186 | No sig change | |  | |
| Knee Flexor Strength | Vogel 1977^d^ | Female | 159 | No sig change | |  | |
|  |  | Male | 186 | No sig change | |  | |
| Upright Pull from 38cm (kg)^c,f^ | Teves 1985^d^ | Female | 487 | 77.1 (13.0) | 95.2 (17.1) | 23.5 |  |
|  |  | Male | 465 | 125.1 (21.2) | 148.6 (24.8) | 18.8 |  |
|  | Sharp 2000^d^ | Female | 99 | 81.7 (19.4) | 85.3 (16.9) | 4.4 |  |
|  |  | Male | 99 | 133.3 (23.6) | 133.2 (22) | -0.1 |  |
|  | Harwood 1999^d^ | Female | 38 | 85.1 (10.5) | 78.2 (10.8) | -8.1 |  |
|  |  | Male | 68 | 137.0 (19.0) | 137.0 (21.0) | 0.0 |  |
|  | Patterson 2005 ^a,d^ | Female int | 9 | 84.7 (14.7) | 93.6 (17.8) | 10.5 |  |
|  |  | Female comp | 3 | 75.0 (11.4) | 107.3 (21.0) | 43.1 |  |
|  |  | Male int | 13 | 140.6 (19.3) | 155.8 (22.8) | 10.8 |  |
|  |  | Male comp | 6 | 146.8 (12.2) | 160.7 (23.4) | 9.5 |  |
|  | Williams 2002 | Female | 8 | 762 (122) | 758 (95) | -0.5 |  |
|  |  | Male | 38 | 1309 (200) | 1244 (196) | -5.0 |  |
|  | Mason 1996^d^ | Female | 20 | 81.1 (NR) | 93 (NR) | 14.7 |  |
|  |  | Male | 22 | 128.1 (NR) | 132.7 (NR) | 3.6 |  |
|  | Daniels 1980^d^ | Female | 7 | 88.1 (3.9) | 87.8 (4.1) | -0.3 |  |
|  |  | Male | 11 | 134.3 (6.4) | 140.9 (5.9) | 4.9 |  |

**Notes:** a, significant differences between females and males at pre-training time point; b, measured as 1 rep max; c, all measured in kg except Patterson and Williams where units are Newtons and the height was not recorded and Daniels 1980, where the height was 132cm); d, measured by dynamometer; e, participants held a semi-squatted position with knees bent and head and back straight, then grasped a fixed bar 38cm high attached via aircraft cable and pulled vertically
**Abbreviations:** comp, comparison; int, intervention; n, number; nr, not reported; sd, standard deviation
**Colour Key:** Dark Green, significantly improved; Dark Red, significantly declined; Light green, improved but not significantly or significance not reported; light red, declined but not significantly or significance not reported

Electronic Supplementary Material Table S7 Grip strength pre to post training

| Activity (units) | Study | Participants | (n) | Pre  mean (sd) | Post  mean (sd) | % Change |  |
| --- | --- | --- | --- | --- | --- | --- | --- |
| Handgrip (kg)^a^ | Teves 1985 | Female | 487 | 30.5 (5.4) | 33.7 (5.6) | 10.5 |  |
|  |  | Male | 465 | 47.5 (7.1) | 52.6 (7.7) | 10.7 |  |
| Combined grip strength (kg) | Jetté 1989 | Female | 68 | 67.4 (8.4) | 66.9 (8.2) | -0.7 |  |
|  |  | Male | 76 | 105.1 (13.7) | 104.9 (14.6) | -0.2 |  |
| Left hand grip strength (N) | von Restorff 2000 | Female | 62 | 322.30 (49.21) | 337.82 (52.40) | 4.8 |  |
|  |  | Male | 48 | 514.82 (81.76) | 534.37 (71.66) | 3.8 |  |
|  | Patterson 2005 | Female int | 9 | 33.3 (6.3) | 32.0 (5.9) | -3.9 |  |
|  |  | Female comp | 3 | 35.3 (4.0) | 35.0 (3.5) | -0.8 |  |
|  |  | Male int | 13 | 52.2 (6.1) | 51.0 (8.0) | -2.3 |  |
|  |  | Male comp | 6 | 55.8 (8.0) | 50.5 (10.2) | -9.5 |  |
| Right hand grip strength (N) | von Restorff 2000 | Female | 62 | 351.02 (47.94) | 368.41 (48.31) | 5.0 |  |
|  |  | Male | 48 | 553.04 (70.08) | 580.97 (72.2.0) | 5.1 |  |
|  | Patterson 2005 | Female int | 9 | 35.9 (6.2) | 34.3 (7.1) | -4.5 |  |
|  |  | Female comp | 3 | 38.0 (3.5) | 37.3 (0.6) | -1.8 |  |
|  |  | Male int | 13 | 52.8 (6.5) | 50.0 (7.4) | -5.3 |  |
|  |  | Male comp | 6 | 58.4 (5.3) | 53.8 (7.7) | -7.9 |  |
| ILM 70-lb hold^b^ | Hart 1985 | Female | 278 | Increase by 60.4 %; P<0.001 | | 60.4 | |
|  |  | Male | 309 | Increase by 5.3 %; P<0.01 | | 5.3 | |

**Notes:** a, one hand, unknown which hand; b, no further information given;
**Colour Key:** Dark Green, significantly improved; Dark Red, significantly declined; Light green, improved but not significantly or significance not reported; light red, declined but not significantly or significance not reported
