# Supplementary material for: Sex-Specific Changes in Physical Performance Following Military Training: A Systematic Review
Source: Sports Med. 2018 Sep 19;48(11):2623–40. doi: 10.1007/s40279-018-0983-4 (PMC6182553; doi:10.1007/s40279-018-0983-4)
Supplement: Supplementary file 2 — Supplementary material 2 (DOCX 246 kb) [file 40279_2018_983_MOESM2_ESM.docx]

Electronic Supplementary Material Appendix S1

Bibliographic Literature searching

| **Database** | **Hits** |
| --- | --- |
| MEDLINE + MEDLINE In Process | 586 |
| EMBASE | 698 |
| CINAHL | 209 |
| HMIC | 12 |
| SPORTDiscus | 155 |
| Web of Science | 1316 |
| PubMed (limited to e-publications) | 165 |
| Total | 3141 |
| - duplicates | 1192 |
| Unique studies to screen | 1949 |

Database: Ovid MEDLINE(R) In-Process & Other Non-Indexed Citations and Ovid MEDLINE(R)

Host: OVID

Data Parameters: 1946 to Present

Date Searched: 23/12/2015

Searcher: Chris Cooper                       Strategy Checked By: Jo Varley-Campbell

Search Strategy:

| **#** | **Searches** | **Results** |
| --- | --- | --- |
| 1 | (military or soldier$ or troop$1 or cadet$ or "armed forces" or "service personnel" or army or navy or naval or marine$1 or commando$ or officer$1 or airforce or "air force" or aircrew or "special forces" or warfighter$ or triservice or "tri service" or triforce or "tri force" or combat).ti,ab,kw. | 147498 |
| 2 | Military Personnel/ | 27340 |
| 3 | 1 or 2 | 156435 |
| 4 | ((man or men or male$1) adj5 (female$1 or women or woman)).ti,ab,kw. | 464869 |
| 5 | (training or conditioning or retrain or "re-train" or "re train" or detrain or "de-train" or "de train" or detraining).ti,ab,kw. | 322896 |
| 6 | *Physical Endurance/ | 9023 |
| 7 | (fit$ adj3 (test$ or training or assessment or standard$ or exam$)).ti,ab,kw. | 10143 |
| 8 | *Physical Fitness/ | 14204 |
| 9 | (((physical or combat) adj3 (fitness or assessment or test$ or exam$ or standard$1 or performance)) or physiological or aerobic or anaerobic).ti,ab,kw. | 531649 |
| 10 | *Exercise Test/ | 15864 |
| 11 | 5 or 6 or 7 or 8 or 9 or 10 | 865103 |
| 12 | 3 and 4 and 11 | 586 |

Notes: N/A

Dataset saved as: MEDLINE586.txt

Database: EMBASE

Host: OVID

Data Parameters: 1974 to 2015 December 22

Date Searched: 23/12/2015

Searcher: Chris Cooper                       Strategy Checked By: Jo Varley-Campbell

Search Strategy:

| **#** | **Searches** | **Results** |
| --- | --- | --- |
| 1 | (military or soldier$ or troop$1 or cadet$ or "armed forces" or "service personnel" or army or navy or naval or marine$1 or commando$ or officer$1 or airforce or "air force" or aircrew or "special forces" or warfighter$ or triservice or "tri service" or triforce or "tri force" or combat).ti,ab,kw. | 174193 |
| 2 | soldier/ | 24727 |
| 3 | army/ | 11500 |
| 4 | air force/ | 1527 |
| 5 | navy/ | 1466 |
| 6 | 1 or 2 or 3 or 4 or 5 | 184921 |
| 7 | ((man or men or male$1) adj5 (female$1 or women or woman)).ti,ab,kw. | 635229 |
| 8 | (training or conditioning or retrain or "re-train" or "re train" or detrain or "de-train" or "de train" or detraining).ti,ab,kw. | 423741 |
| 9 | *endurance/ | 5920 |
| 10 | (fit$ adj3 (test$ or training or assessment or standard$ or exam$)).ti,ab,kw. | 12664 |
| 11 | *fitness/ | 13653 |
| 12 | (((physical or combat) adj3 (fitness or assessment or test$ or exam$ or standard$1 or performance)) or physiological or aerobic or anaerobic).ti,ab,kw. | 682095 |
| 13 | *Exercise Test/ | 12574 |
| 14 | 8 or 9 or 10 or 11 or 12 or 13 | 1105462 |
| 15 | 6 and 7 and 14 | 698 |

Notes: N/A

Dataset saved as: Embase698.txt

Database: CINAHL

Host: EBSCOhost

Data Parameters: 1961-Present

Date Searched: 23/12/2015

Searcher: Chris Cooper                       Strategy Checked By: Jo Varley-Campbell
Search Strategy:

| **#** | **Query** | **Limiters/Expanders** | **Last Run Via** | **Results** |
| --- | --- | --- | --- | --- |
| S12 | S3 AND S4 AND S11 | Search modes - Boolean/Phrase | Interface - EBSCOhost Research Databases  Search Screen - Advanced Search  Database - CINAHL Plus with Full Text | 209 |
| S11 | S5 OR S6 OR S7 OR S8 OR S9 OR S10 | Search modes - Boolean/Phrase | Interface - EBSCOhost Research Databases  Search Screen - Advanced Search  Database - CINAHL Plus with Full Text | 146,582 |
| S10 | (MM "Exercise Test") | Search modes - Boolean/Phrase | Interface - EBSCOhost Research Databases  Search Screen - Advanced Search  Database - CINAHL Plus with Full Text | 2,725 |
| S9 | TI ( (((physical or combat) N2 (fitness or assessment or test* or exam* or standard* or performance)) or physiological or aerobic or anaerobic) ) OR AB ( (((physical or combat) N2 (fitness or assessment or test* or exam* or standard* or performance)) or physiological or aerobic or anaerobic) ) | Search modes - Boolean/Phrase | Interface - EBSCOhost Research Databases  Search Screen - Advanced Search  Database - CINAHL Plus with Full Text | 42,626 |
| S8 | (MM "Physical Fitness") | Search modes - Boolean/Phrase | Interface - EBSCOhost Research Databases  Search Screen - Advanced Search  Database - CINAHL Plus with Full Text | 7,405 |
| S7 | TI ( (fit* N2 (test* or training or assessment or standard* or exam*)) ) OR AB ( (fit* N2 (test* or training or assessment or standard* or exam*)) ) | Search modes - Boolean/Phrase | Interface - EBSCOhost Research Databases  Search Screen - Advanced Search  Database - CINAHL Plus with Full Text | 2,841 |
| S6 | (MM "Physical Endurance") | Search modes - Boolean/Phrase | Interface - EBSCOhost Research Databases  Search Screen - Advanced Search  Database - CINAHL Plus with Full Text | 2,246 |
| S5 | TI ( (training or conditioning or retrain or "re-train" or "re train" or detrain or "de-train" or "de train" or detraining) ) OR AB ( (training or conditioning or retrain or "re-train" or "re train" or detrain or "de-train" or "de train" or detraining) ) | Search modes - Boolean/Phrase | Interface - EBSCOhost Research Databases  Search Screen - Advanced Search  Database - CINAHL Plus with Full Text | 100,337 |
| S4 | TI ( ((man or men or male*) N4 (female* or women or woman)) ) OR AB ( ((man or men or male*) N4 (female* or women or woman)) ) | Search modes - Boolean/Phrase | Interface - EBSCOhost Research Databases  Search Screen - Advanced Search  Database - CINAHL Plus with Full Text | 67,760 |
| S3 | S1 OR S2 | Search modes - Boolean/Phrase | Interface - EBSCOhost Research Databases  Search Screen - Advanced Search  Database - CINAHL Plus with Full Text | 30,443 |
| S2 | (MH "Military Personnel+") | Search modes - Boolean/Phrase | Interface - EBSCOhost Research Databases  Search Screen - Advanced Search  Database - CINAHL Plus with Full Text | 11,276 |
| S1 | TI ( (military or soldier* or troop* or cadet* or "armed forces" or "service personnel" or army or navy or naval or marine* or commando* or officer* or airforce or "air force" or aircrew or "special forces" or warfighter* or triservice or "tri service" or triforce or "tri force" or combat) ) OR AB ( (military or soldier* or troop* or cadet* or "armed forces" or "service personnel" or army or navy or naval or marine* or commando* or officer* or airforce or "air force" or aircrew or "special forces" or warfighter* or triservice or "tri service" or triforce or "tri force" or combat) ) | Search modes - Boolean/Phrase | Interface - EBSCOhost Research Databases  Search Screen - Advanced Search  Database - CINAHL Plus with Full Text | 26,394 |

Notes: N/A

Dataset saved as: CINAHL209.txt

Database: HMIC

Host: OVID

Data Parameters: 1979 to November 2015

Date Searched: 23/12/2015

Searcher: Chris Cooper                       Strategy Checked By: Jo Varley-Campbell
Search Strategy:

| **#** | **Searches** | **Results** |
| --- | --- | --- |
| 1 | (military or soldier$ or troop$1 or cadet$ or "armed forces" or "service personnel" or army or navy or naval or marine$1 or commando$ or officer$1 or airforce or "air force" or aircrew or "special forces" or warfighter$ or triservice or "tri service" or triforce or "tri force" or combat).ti,ab. | 5471 |
| 2 | armed forces personnel/ | 211 |
| 3 | armed forces/ | 213 |
| 4 | 1 or 2 or 3 | 5627 |
| 5 | ((man or men or male$1) adj5 (female$1 or women or woman)).ti,ab. | 5025 |
| 6 | (training or conditioning or retrain or "re-train" or "re train" or detrain or "de-train" or "de train" or detraining).ti,ab. | 19521 |
| 7 | (fit$ adj3 (test$ or training or assessment or standard$ or exam$)).ti,ab. | 145 |
| 8 | physical fitness/ | 176 |
| 9 | (((physical or combat) adj3 (fitness or assessment or test$ or exam$ or standard$1 or performance)) or physiological or aerobic or anaerobic).ti,ab. | 1210 |
| 10 | 6 or 7 or 8 or 9 | 20861 |
| 11 | 4 and 5 and 10 | 12 |

Notes: N/A

Dataset saved as: HMIC12.txt

Database: SPORTDiscus

Host: EBSCOhost

Data Parameters: 1949-Present

Date Searched: 23/12/2015

Searcher: Chris Cooper                       Strategy Checked By: Jo Varley-Campbell
Search Strategy:

| **#** | **Query** | **Limiters/Expanders** | **Last Run Via** | **Results** |
| --- | --- | --- | --- | --- |
| S8 | S1 AND S2 AND S7 | Search modes - Boolean/Phrase | Interface - EBSCOhost Research Databases  Search Screen - Advanced Search  Database - SPORTDiscus | 155 |
| S7 | S3 OR S4 OR S5 OR S6 | Search modes - Boolean/Phrase | Interface - EBSCOhost Research Databases  Search Screen - Advanced Search  Database - SPORTDiscus | 232,780 |
| S6 | TI ( (((physical or combat) N2 (fitness or assessment or test* or exam* or standard* or performance)) or physiological or aerobic or anaerobic) ) OR AB ( (((physical or combat) N2 (fitness or assessment or test* or exam* or standard* or performance)) or physiological or aerobic or anaerobic) ) | Search modes - Boolean/Phrase | Interface - EBSCOhost Research Databases  Search Screen - Advanced Search  Database - SPORTDiscus | 54,000 |
| S5 | DE "PHYSICAL fitness" OR DE "ANAEROBIC exercises" OR DE "ASTROLOGY & physical fitness" OR DE "BODYBUILDING" OR DE "CARDIOVASCULAR fitness" OR DE "CIRCUIT training" OR DE "COMPOUND exercises" OR DE "EXERCISE tolerance" OR DE "ISOLATION exercises" OR DE "LIANGONG" OR DE "MUSCLE strength" OR DE "PERIODIZATION training" OR DE "PHYSICAL fitness -- Genetic aspects" OR DE "PHYSICAL fitness for children" OR DE "PHYSICAL fitness for girls" OR DE "PHYSICAL fitness for men" OR DE "PHYSICAL fitness for older people" OR DE "PHYSICAL fitness for people with disabilities" OR DE "PHYSICAL fitness for women" OR DE "PHYSICAL fitness for youth" OR DE "SPORT for all" | Search modes - Boolean/Phrase | Interface - EBSCOhost Research Databases  Search Screen - Advanced Search  Database - SPORTDiscus | 100,650 |
| S4 | TI ( (fit* N2 (test* or training or assessment or standard* or exam*)) ) OR AB ( (fit* N2 (test* or training or assessment or standard* or exam*)) ) | Search modes - Boolean/Phrase | Interface - EBSCOhost Research Databases  Search Screen - Advanced Search  Database - SPORTDiscus | 5,940 |
| S3 | TI ( (training or conditioning or retrain or "re-train" or "re train" or detrain or "de-train" or "de train" or detraining) ) OR AB ( (training or conditioning or retrain or "re-train" or "re train" or detrain or "de-train" or "de train" or detraining) ) | Search modes - Boolean/Phrase | Interface - EBSCOhost Research Databases  Search Screen - Advanced Search  Database - SPORTDiscus | 125,254 |
| S2 | TI ( ((man or men or male*) N4 (female* or women or woman)) ) OR AB ( ((man or men or male*) N4 (female* or women or woman)) ) | Search modes - Boolean/Phrase | Interface - EBSCOhost Research Databases  Search Screen - Advanced Search  Database - SPORTDiscus | 38,530 |
| S1 | TI ( (military or soldier* or troop* or cadet* or "armed forces" or "service personnel" or army or navy or naval or marine* or commando* or officer* or airforce or "air force" or aircrew or "special forces" or warfighter* or triservice or "tri service" or triforce or "tri force" or combat) ) OR AB ( (military or soldier* or troop* or cadet* or "armed forces" or "service personnel" or army or navy or naval or marine* or commando* or officer* or airforce or "air force" or aircrew or "special forces" or warfighter* or triservice or "tri service" or triforce or "tri force" or combat) ) | Search modes - Boolean/Phrase | Interface - EBSCOhost Research Databases  Search Screen - Advanced Search  Database - SPORTDiscus | 28,419 |

Notes: N/A

Dataset saved as: SPORTDiscus155.txt

Database: Web of Science (SCI, SSCi, CPC-I, CPCI-SSH, and ESCI)

Host: ISI (Thomson Reuters)

Data Parameters: 1900-2015

Date Searched: 23/12/2015

Searcher: Chris Cooper                       Strategy Checked By: Jo Varley-Campbell
Search Strategy:


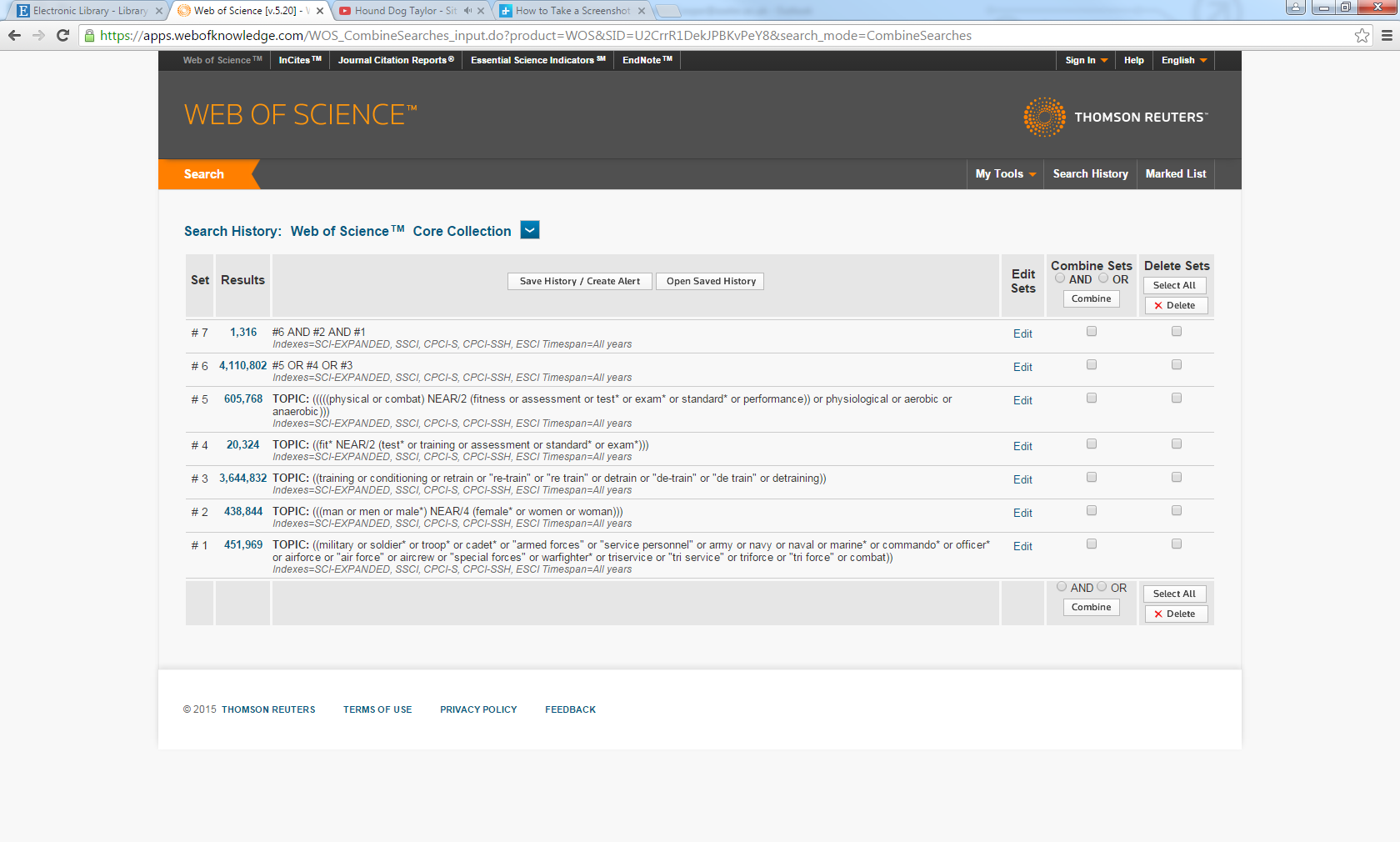


Notes: N/A

Dataset saved as: WOS1316.txt

Database: PubMed

Host: National Library of Medicine (<http://www.ncbi.nlm.nih.gov/pubmed> )

Data Parameters: 1946 to Present

Date Searched: 23/12/2015

Searcher: Chris Cooper

Search Strategy:


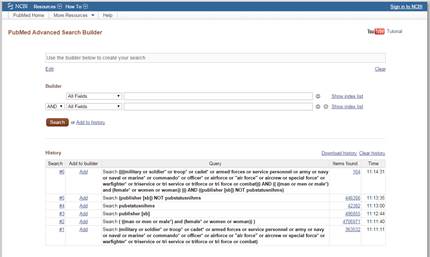


Notes: N/A

Dataset saved as: PubMed165.txt

Supplementary searching

The following supplementary searches were undertaken in SCOPUS (Elsevier) on 13/01/2016 and 14/01/2016.

Table A Supplementary Searching of Included Studies

| **N** | **Citation** | **Forwards**  **Chase** | **Backwards**  **Chase** | **First**  **Author** | **Last**  **Author** | **Contacted** |
| --- | --- | --- | --- | --- | --- | --- |
| 1 | Knapik, J. J., Hauret, K. G., Arnold, S., Canham-Chervak, M., Mansfield, A. J., Hoedebecke, E. L., & McMillian, D. (2003). *Injury and fitness outcomes during implementation of physical readiness training*. Army Center For Health Promotion And Preventive Medicine Aberdeen Proving Ground MD. | 0  (although the study linked to this report generated n=36, so these were chased) | 6 | 135 | 6 | Yes |
| 2 | Knapik, J., Darakjy, S., Scott, S. J., Hauret, K. G., MARIN, R., Rieger, W., & Jones, B. H. (2004). Evaluation of a standardized physical training program for basic combat training. *The Journal of Strength & Conditioning Research*,*19*(2), 246-253. | 29 | 3 | (already chased at #1) | 127 | Yes |
| 3 | Knapik, J. J., Wright, J. E., Kowal, D. M., & Vogel, J. A. (1980). *The influence of US Army basic initial training on the muscular strength of men and women*(No. USARIEM-M-11/80). Army research inst of environmental medicine natick MA. | Not identified in SCOPUS | 0 | (already chased at #1) | 63 (as first) | Yes (at Knpaick 03) |
| 4 | Teves MA, Wright JE and Vogel JA. Performance on selected candidate screening test procedures before and after Army basic and advanced individual training. Natick, MA: USARIEM Technical Report 13/85, June 1985 | Not identified in SCOPUS | 0 | Not identified in SCOPUS | (already chased at #3) | Pre-1999 study |
| 5 | Bell, N. S., Mangione, T. W., Hemenway, D., Amoroso, P. J., & Jones, B. H. (2000). High injury rates among female army trainees: a function of gender? *American journal of preventive medicine*, *18*(3), 141-146. | 71 | 1 | 43 | (already chased at #2) | Yes |
| 6 | Hart, L. E. M., Allen, C. L., & Cox, K. M. (1985). Effect of a 10-week military training program on upper body strength in male and female recruits. *Medicine & Science in Sports & Exercise*, *17*(2), 195. | Not identified in SCOPUS | Not identified in SCOPUS | Not identified in SCOPUS | Not identified in SCOPUS | Pre-1999 study |
| 7 | Sharp, M. A., Knapik, J. J., Patton, J. F., Smutok, M. A., Hauret, K., Chervak, M., . . . Jones, B. H. (2000). Physical fitness of soldiers entering and leaving basic combat training. (Report No. T00-13). Natick, MA: U.S. Army Research Institute of Environmental Medicine. | Not identified in SCOPUS | 2  (1 =ex4  1=teves) | 50 | (already chased at #2) | Yes |
| 8 | Vogel, J. A., Ramos, M. U., Patton, J. F., & Vogel, J. A. (1977). Comparisons of aerobic power and muscle strength between men and women entering the US ARMY. *Medicine & Science in Sports & Exercise*, *9*(1), 58. | 0 | 0 | (already chased at #3) | N/A  (reported as a sole author study) | Pre-1999 study |
| 9 | Yanovich, R., Merkel, D., Israeli, E., Evans, R. K., Erlich, T., & Moran, D. S. (2011). Anemia, iron deficiency, and stress fractures in female combatants during 16 months. *The Journal of Strength & Conditioning Research*, *25*(12), 3412-3421. | 2 | 6 | 30 | 132 | Yes |
| 10 | Evans, R. K., Antczak, A. J., Lester, M., Yanovich, R., Israeli, E. R. A. N., & Moran, D. S. (2008). Effects of a 4-month recruit training program on markers of bone metabolism. *Medicine and science in sports and exercise*, *40*(11 Suppl), S660-70. | 29 | 0 | 31 | (already chased at #9) | Yes |
| 11 | Jetté, M., Sidney, K., & Kimick, A. (1989). Effects of basic training on Canadian forces recruits. *Canadian journal of sport sciences= Journal canadien des sciences du sport*, *14*(3), 164-172. | 3 | 0 | 71 | 3 | Pre-1999 study |
| 12 | Patton, J. F., Daniels, W. L., & Vogel, J. A. (1980). Aerobic power and body fat of men and women during army basic training. Aviation, space, and environmental medicine, 51(5), 492-496. | 35 | 0 | 65 | (already chased at #3) | Pre-1999 study |
| 13 | Wood, P. S., & Kruger, P. E. (2013). Comparison of physical fitness outcomes of young South African military recruits following different physical training programs during basic military training. *South African Journal for Research in Sport, Physical Education and Recreation*, *35*(1), 203-217. | Not identified in SCOPUS | 1 | 6 | 13 | Yes |
| 14 | Drain, J. R., Sampson, J. A., Billing, D. C., Burley, S. D., Linnane, D. M., & Groeller, H. (2015). The Effectiveness of Basic Military Training To Improve Functional Lifting Strength in New Recruits. *The Journal of Strength & Conditioning Research*, *29*, S173-S177. | 0 | 1 | 3 | 21 | Yes |
| 15 | Sonna, L. A., Sharp, M. A., Knapik, J. J., Cullivan, M., Angel, K. C., Patton, J. F., & Lilly, C. M. (2001). Angiotensin-converting enzyme genotype and physical performance during US Army basic training. *Journal of Applied Physiology*, *91*(3), 1355-1363. | 39 | 6 | 29 | 133 | Yes |
| 16 | Yanovich, R., Evans, R., Israeli, E., Constantini, N., Sharvit, N., Merkel, D., ... & Moran, D. S. (2008). Differences in physical fitness of male and female recruits in gender-integrated army basic training. *Medicine and science in sports and exercise*, *40*(11 Suppl), S654-9. | 7 | 0 | (already chased at #3) | (already chased at #9) | Yes |
| 17 | Restorff, W. V. (2000). Physical fitness of young women: carrying simulated patients. *Ergonomics*, *43*(6), 728-743. | 12 | 2 | 2 | N/A  Sole author study | Pre-1999 study |
| 18 | Harwood, G. E. (1999). Fitness, performance, and risk of injury in British Army Officer Cadets. *Military medicine*, *164*(428), 428. | 19 | 4 | 1 | 324 | Yes |
| 19 | Sharp, D. S., Wright, J. E., Vogel, J. A., Patton, J. F., Daniel, W. L., Knapik, J., & Korval, D. M. (1980). *Screening for physical capacity in the U.S. Army: An analysis of measures predictive of strength and stamina.* (Report No. T8/80). Natick, MA: U.S. Army Research Institute of Environmental Medicine. | Not identified in SCOPUS | 0 | Not identified in SCOPUS | Not identified in SCOPUS | Pre-1999 study |
| 20 | Rayson, Wilkinson, Valk and Nevill. (2003) The physical demands of army basic training. Contemporary ergonomics | Not identified in SCOPUS | 1 (ex7) | 48 | Not identified in SCOPUS | Yes |
| 21 | Patterson, MJ, Roberts, WS, and Lau, WM. Gender and physical training effects on soldier physical competencies and physiological strain. Fishermans Bend, Victoria, Australia: Australian Defense Science and Technology Organization. Technical Report No. DSTO-TR-1875, 2005 | Not identified in SCOPUS | 0 | Not identified in SCOPUS | Not identified in SCOPUS | No* |
| 22 | Richmond, V. L., Carter, J. M., Wilkinson, D. M., Horner, F. E., Rayson, M. P., Wright, A., & Bilzon, J. L. (2012). Comparison of the physical demands of single-sex training for male and female recruits in the British Army. *Military medicine*, *177*(6), 709-715. | 2 | 3 | 20 | (already chased at #20) | No* |
| 23 | Daniels, W. L., Kowal, D. M., Vogel, J. A., & Stauffer, R. M. (1979). Physiological effects of a military training program on male and females cadets Aviation, space, and environmental medicine, 50(6), 562-6. | 18 | 0 | 14 | 1 | Pre-1999  study |
| 24 | Mason, M. J., N’jie, M., Holliman, D. E., & Rayson, M. P. (1996). The physical demands of basic training in British Army recruits: A pilot study.*DRA/CHS (HS2)/CR96/019. Ministry of Defence*. | Not identified in SCOPUS | 0 | Not identified in SCOPUS | (already chased at #20) | Pre-1999  study |
| 25 | Marcinik, E. J., & Hodgdon, J. A. (1984, January). Shipboard physical conditioning-a pilot-study of circuit weight training for navy men and women. *In* *aviation space and environmental medicine* (Vol. 55, No. 5, pp. 463-463). 320 S Henry St, Alexandria, VA 22314-3579: Aerospace Medical Assoc. | Not identified in SCOPUS | 0 | Not identified in SCOPUS | Not identified in SCOPUS | Pre-1999 study |
| 26 | Blacker, S. D., Wilkinson, D. M., & Rayson, M. P. (2009). Gender differences in the physical demands of British Army recruit training. *Military medicine*, *174*(8), 811-816. | 6 | 9 | 30 | (already chased at #20) | No* |
| 27 | Daniels, W. L., Wright, J. E., Sharp, D. S., Kowal, D. M., & Mello, R. P. (1980). *The Effect of Two Years Training on Aerobic Power and Muscle Strength of Male and Female Cadets* (No. USARIEM-M-12/80). Army research inst of environmental medicine natick ma. | Not identified in SCOPUS | 1 | (already chased at #14) | 26 | Pre-1999 study |
| 28 | GAMBERA, C. P. J., Schneeman, B. O., & Davis, P. A. (1995). Use of the Food Guide Pyramid and US Dietary Guidelines to improve dietary intake and reduce cardiovascular risk in active-duty Air Force members. *Journal of the American Dietetic Association*, *95*(11), 1268-1273. | 21 | 0 | 1 | 171 | Pre-1999 study |
| 29 | A.G. Williams , M.P. Rayson & D.A. Jones (2002) Resistance training and the enhancement of the gains in material-handling ability and physical fitness of British Army recruits during basic training, Ergonomics, 45:4, 267-279 | 20 | 0 | 65 | 288 | No* |
|  | Totals | 349 | 46 | 594 | 1358 |  |

* 4 studies were identified after data cut-off.

Web searching

Table B Web Searching

| **Date** | **Portal/URL** | **Search Terms** | **Results**  **(checked/included)** | **Notes** |
| --- | --- | --- | --- | --- |
| 12/01/16 | Dogpile  <http://www.dogpile.com/> | ((Physical training) and (man or men or male or males) and (female or females or women or woman) and (military)) | 4/0  NO INCLUDES | First 5 pages screened on title (n=50 records)  4 records checked, all EX1 |
| 12/01/16 | Google  <https://www.google.co.uk/webhp?hl=en> | ((Physical training) and (man or men or male or males) and (female or females or women or woman) and (military)) | 1. Sharp 1994;  2. English March April 2015 Military Review Eden - MilitaryReview_20150430_art009  NO INCLUDES | First 5 pages screened on title (n=50 records)  All studies EX1 |
| 12/01/16 | Google Advanced Search + file type limit to PDF  <https://www.google.co.uk/webhp?hl=en> | ((Physical training) and (man or men or male or males) and (female or females or women or woman) and (military)) | 1. Sharp 1993  NO INCLUDES | First 5 pages screened on title (n=50 records)  All studies EX1 |
| 12/01/16 | OpenGrey  <http://www.opengrey.eu/> | 1. ((Physical training) and (man or men or male or males) and (female or females or women or woman) and (military))  2. military training keyword:(WOMAN)  3. Physical training and military | 1. n=0  2. n=5 (5/0)  3. n=15 (15/1)  NO INCLUDES | Search 3 – record was ex1 |
| 12/01/16 | British Library Main Catalogue  <http://explore.bl.uk/primo_library/libweb/action/search.do?vid=BLVU1> | 1. ((Physical training) and (man or men or male or males) and (female or females or women or woman) and (military)) | 1. n=26 (3/0)  NO INCLUDES | All duplicates of main search |
| 30/01/2016 | Defense Technical Information Center  <http://www.dtic.mil/dtic/> | (((male) and (female)) AND (training)) First 50 records screened.  (((men) and (women)) and (training)) First 50 records screened. | N=7 taken forward to full-text screening  N=2 taken forward to full-text screening |  |

The Women in the GCC Team undertook searches of Athena (the DSTL database) on 22/01/2016. The following search terms were used. No studies that uniquely met the review’s inclusion criteria were identified.

(((male) and (female)) AND (training));

(((men) and (women)) and (training));

(((men) and (women)) and (muscle));

(((male) and (female)) AND (muscle));

(((male) and (female)) AND (aerobic));

(((men) and (women)) AND (aerobic));

(((male) and (female)) AND (Anaerobic));

(((men) and (women)) AND (Anaerobic));

(((male) and (female)) AND (Detraining));

(((men) and (women)) AND (Detraining));

(((male) and (female)) AND (Injury));

(((men) and (women)) AND (Injury)).

Table C Identified studies by database

Bethel A, Rogers M 2015 *Search summary table for Systematic Reviews (SRs)* Cochrane Colloquium, Vienna

| **Project title: Optimal Physical Training Strategies to Prepare Male and Female Military Personnel for Performance of Tasks Associated with Ground Close Combat (GCC) Roles** | | | | | | | | | | | | | | |
| --- | --- | --- | --- | --- | --- | --- | --- | --- | --- | --- | --- | --- | --- | --- |
| Included references | **Database searches** | | | | | | | | | | **Supplementary searches** | | | |
|  | **MEDLINE** | **EMBASE** | **CINAHL** | **HMIC** | **SPORTDiscus** | **Web of Science** | **PubMed** | **d/b 8** | **d/b 9** | **d/b 10** | **fcs** | **bcs** | **ac** | **wss** |
| Knapik 03 |  |  |  |  |  |  |  |  |  |  | X | X | X |  |
| Knapik 04 | X |  | X |  | X | X |  |  |  |  | X | X | X |  |
| Knapik 80 | X |  |  |  |  | X |  |  |  |  |  |  | X | X |
| Teves 85 |  |  |  |  |  |  |  |  |  |  |  | X |  | X |
| Bell 2000 |  |  |  |  |  | X |  |  |  |  | X |  | X |  |
| Hart 1985 |  |  |  |  |  | X |  |  |  |  |  |  |  |  |
| Sharp MA 00 |  |  |  |  |  |  |  |  |  |  |  |  |  | X |
| Vogel 77 |  |  |  |  |  | X |  |  |  |  |  |  |  |  |
| Yanovich11 | X |  | X |  |  | X |  |  |  |  | X |  | X |  |
| Evans 08 | X |  | X |  |  | X |  |  |  |  |  |  | X |  |
| Jetté 89 | X |  |  |  |  |  |  |  |  |  |  |  | X |  |
| Patton 80 | X |  |  |  | X | X |  |  |  |  |  |  | X |  |
| Wood 13 |  |  |  |  | X | X |  |  |  |  |  |  |  |  |
| Drain 15 | X |  | X |  | X |  |  |  |  |  |  |  |  |  |
| Sonna 01 | X |  |  |  |  | X |  |  |  |  | X |  | X |  |
| Yanovich 08 | X |  | X |  | X | X |  |  |  |  |  |  | X |  |
| Restorff 00 | X | X |  |  |  |  |  |  |  |  |  |  | X |  |
| Harwood 99 |  |  |  |  |  | X |  |  |  |  |  |  | X |  |
| Sharp 80 |  |  |  |  |  |  |  |  |  |  |  | X |  |  |
| Rayson 03 |  |  |  |  |  | X |  |  |  |  |  |  |  |  |
| Patterson 05 |  |  |  |  |  |  |  |  |  |  |  | X | X |  |
| Richmond 12 | X |  | X |  |  | X |  |  |  |  | X | X | X |  |
| Daniels 79 | X |  |  |  |  | X |  |  |  |  |  |  | X |  |
| Mason 96 |  |  |  |  |  |  |  |  |  |  |  | X |  |  |
| Marcinik 84 |  |  |  |  |  | X |  |  |  |  |  |  |  |  |
| Blacker 09 |  |  |  |  |  |  |  |  |  |  | X | X | X |  |
| Daniels 80 |  |  |  |  |  |  |  |  |  |  | X |  |  | X |
| Gambera 95 | X |  |  |  |  | X |  |  |  |  |  | X |  |  |
| Wiliams 02 |  |  |  |  |  |  |  |  |  |  | X | X | X |  |
| No. included refs | 13 | 1 | 3 | 0 | 5 | 17 | 0 |  |  |  | 9 | 10 | 18 | 3 |
| No. unique refs |  |  |  |  |  | 2 |  |  |  |  | 1 | 1 | 1 |  |
